# Lineage-Specific Genes Are Prominent DNA Damage Hotspots during Leukemic Transformation of B Cell Precursors

**DOI:** 10.1016/j.celrep.2017.01.057

**Published:** 2017-02-14

**Authors:** Bryant Boulianne, Mark E. Robinson, Philippa C. May, Leandro Castellano, Kevin Blighe, Jennifer Thomas, Alistair Reid, Markus Müschen, Jane F. Apperley, Justin Stebbing, Niklas Feldhahn

**Affiliations:** 1 Centre for Haematology, Department of Medicine, Imperial College London, W12 0NN London, UK; 2 Division of Cancer, Department of Surgery and Cancer, Imperial College London, W12 0NN London, UK; 3 Molecular Pathology, Imperial College Healthcare NHS Trust, W12 0NN London, UK; 4 Department of Systems Biology, Beckman Research Institute and City of Hope Comprehensive Cancer Center, Pasadena, CA 91016, USA

**Keywords:** leukemia, B-cell precursors, transformation, oncogenic stress, DNA damage, genomic instability, clonal evolution, transcription

## Abstract

In human leukemia, lineage-specific genes represent predominant targets of deletion, with lymphoid-specific genes frequently affected in lymphoid leukemia and myeloid-specific genes in myeloid leukemia. To investigate the basis of lineage-specific alterations, we analyzed global DNA damage in primary B cell precursors expressing leukemia-inducing oncogenes by ChIP-seq. We identified more than 1,000 sensitive regions, of which B lineage-specific genes constitute the most prominent targets. Identified hotspots at B lineage genes relate to DNA-DSBs, affect genes that harbor genomic lesions in human leukemia, and associate with ectopic deletion in successfully transformed cells. Furthermore, we show that most identified regions overlap with gene bodies of highly expressed genes and that induction of a myeloid lineage phenotype in transformed B cell precursors promotes de novo DNA damage at myeloid loci. Hence, we demonstrate that lineage-specific transcription predisposes lineage-specific genes in transformed B cell precursors to DNA damage, which is likely to promote the frequent alteration of lineage-specific genes in human leukemia.

## Highlights

• B lineage genes are γH2AX-marked DNA damage sites in transformed B cell precursors
• γH2AX hotspots occur at highly transcribed, H3K27ac-marked genes
• γH2AX hotspots relate to R loop sequences and con/divergent transcription
• Myeloid lineage induction in transformed B cells causes DNA damage at myeloid loci

## Graphical Abstract

**Figure:**
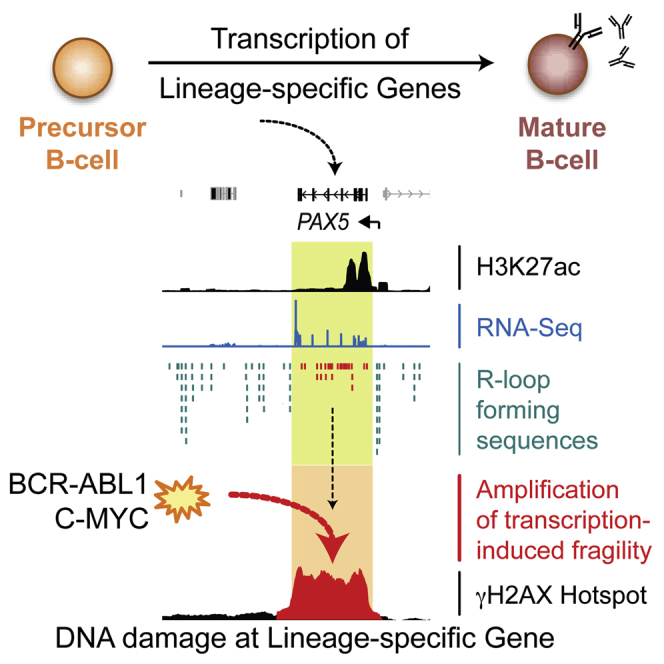

## Teaser

B lineage-specific genes are frequently altered in B lymphoid leukemia. Boulianne et al. show that transformed B cell precursors exhibit transcription-coupled DNA damage at B lineage genes. Likewise, lymphoid-to-myeloid conversion of transformed B cells induces DNA damage at myeloid loci, suggesting that lineage-specific transcription predisposes lineage-specific genes to subsequent alterations as observed in human leukemia.

## Introduction

Most cancers are genetically unstable (Lengauer et al., 1998) and exhibit genomic alterations that promote tumor progression (Vogelstein et al., 2013). Analyses of cancer genomes over the last decade have revealed highly mutated genomes with more than 100 alterations per tumor genome for solid tumors (Vogelstein et al., 2013), whereas liquid tumors such as leukemia are less mutated (Mullighan et al., 2007). The analyses of leukemia genomes, however, uncovered that the most prominent group of genes to become defective in B cell precursor leukemia (B-cell precursor acute lymphoblastic leukemia [BCP-ALL] or B-lymphoid acute lymphoblastic leukemia [B-ALL]) are regulators of B cell development (40% in B-ALL and 66%–68% in high-risk B-ALL (Mullighan et al., 2007, Mullighan et al., 2009, Zhang et al., 2011)), with the transcription factors *PAX5* and *IKZF1* being altered in ∼32% and ∼29% of cases in high-risk B-ALL, respectively (Mullighan et al., 2009). Deletion of IKZF1 is associated with particularly poor clinical outcomes (Mullighan et al., 2009), and recent studies based on a PAX5 restoration model clearly validated the powerful tumor-suppressive function of PAX5 in B lineage ALL (Liu et al., 2014). Similar to lymphoid malignancies, myeloid-lineage genes are often targets of genomic alteration in myeloid leukemia (e.g., *CEBPA*, *GATA1*, *PU.1*, and *RUNX1*; Rosenbauer and Tenen, 2007). Although this pattern of lineage-specific genetic lesions in lymphoid and myeloid leukemia cases has been frequently observed, the mechanistic basis underlying its appearance remained elusive.

DNA damage resulting from oncogenic stress was proposed as a cause of genome instability in transformed cells almost 10 years ago (Halazonetis et al., 2008). Malignant transformation of healthy cells resulting in cancer is frequently associated with the activation of an oncogene (e.g., *BCR-ABL1* in B-ALL), and, similarly, oncogene activation can occur during tumor progression. Oncogene activation may cause cellular stress, including DNA damage, as a result of oxidative stress (Sattler et al., 2000, Vafa et al., 2002) and/or replicative stress (Di Micco et al., 2006). The latter has been associated with hyper-replication, aberrant replication origin firing, and an exhaustion of the cellular nucleotide pool that interferes with efficient replication (Bester et al., 2011). DNA damage in oncogene-expressing cells has been shown to promote malignant transformation and acquisition of genomic defects in cancer models (Gilad et al., 2010). Likewise, oncogene-induced DNA damage has been associated with genomic defects found in human cancer, including solid tumors (Miron et al., 2015) and B cell lymphoma (Barlow et al., 2013). However, the potential relevance of oncogenic stress in promoting genomic defects found in patients with leukemia remained unclear and has been further brought into question by the identification of the antibody-diversifying enzymes AID and RAG1 as potential drivers of genomic defects in ETV6-RUNX1-positive B cell precursor ALL (Swaminathan et al., 2015). Likewise, recurrent genomic deletions in B-ALL have been associated with aberrant RAG1 activity by others because of the presence of cryptic RAG1 cleavage sites at individual deletion breakpoints (Holmfeldt et al., 2013, Iacobucci et al., 2009, Mullighan et al., 2008).

To obtain insight into the origin of DNA damage that promotes genomic defects found in leukemia genomes, here we performed a genome-wide analysis of DNA damage in primary B cell precursors undergoing oncogene-induced leukemic transformation using chromatin immunoprecipitation sequencing (ChIP-seq). The cells analyzed by us do not express AID or RAG1; hence, identified DNA damage is thought to relate primarily to oncogenic stress present in these transformed cells.

## Results

### Transformed B Cell Precursors Exhibit Genome-wide DNA Damage

**Figure 1:**
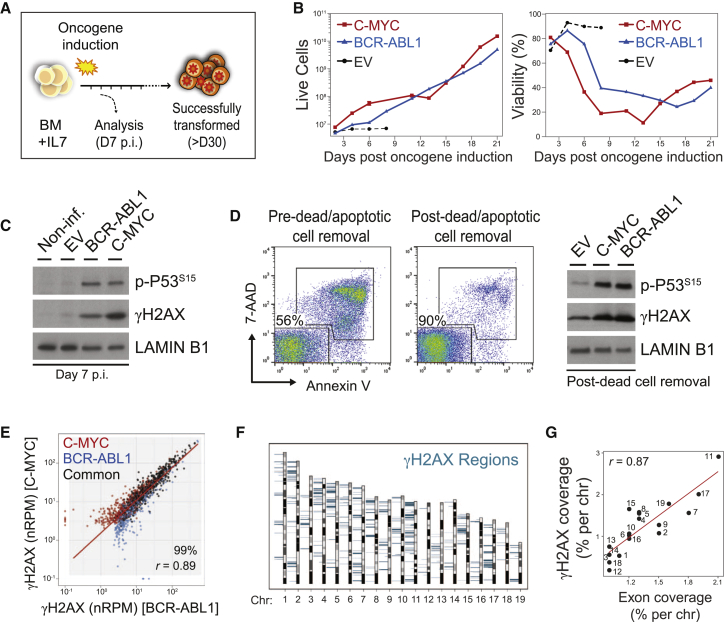
Transformed B Cell Precursors Exhibit Genome-wide DNA Damage (A) Schematic of the experimental strategy. (B) Diagram visualizing proliferation (left) and viability (right) of B cell precursor cultures transduced with the oncogenes BCR-ABL1 or C-MYC or empty vector (EV) as a control. Shown is one of more than five repeat experiments. (C) Western blots for the DNA damage-associated phosphorylations γH2AX and p-P53^Ser15^ in transformed versus untransformed B cell precursors. Shown is one of more than five repeat experiments. (D) Left: verification of the dead cell removal procedure by flow cytometry (7-AAD = dead cells, AnnexinV = apoptotic). Right: western blot as in (C) for dead/apoptotic cell-depleted fractions. (E) Scatterplot of normalized γH2AX read counts per million reads (nRPM, normalized to input/isotype control library) within γH2AX regions identified for BCR-ABL1-expressing cells (blue), C-MYC-expressing cells (red), or for both ChIP-seq libraries (common regions = black) (for repeat experiments, see Figures S1D and S1E). The shaded area indicates regions with γH2AX enrichment above background under both conditions (percent of total regions indicated). Linear regression (red line) and Pearson’s correlation (p < 2.2 × 10^−16^) were calculated from these common regions. (F) Chromosomal distribution of γH2AX regions (blue bars) present in transformed B cell precursors. Bar height represents γH2AX signals (RPM of the BCR-ABL1 library) for each region (range = 0–100 RPM). (G) Comparison of γH2AX region versus exon coverage per chromosome of combined γH2AX regions for BCR-ABL1- and C-MYC-expressing cells. The red line represents linear regression fit.

B lymphoid leukemia arises from hematopoietic stem cells or B cell precursors that acquired a transforming, leukemia-initiating event. To analyze DNA damage related to leukemic transformation, we transduced primary murine bone marrow-derived B cell precursors with retroviral vectors encoding the leukemia-inducing oncogenes BCR-ABL1 or C-MYC or with an empty vector (EV) as a control (Figure 1A; Figure S1A). To increase the sensitivity of DNA damage detection, we used bone marrow from *53BP1*^−/−^ mice. As expected, oncogene expression resulted in an immediate response characterized by elevated proliferation and a temporary phase of cellular stress (Figure 1B). Early-transformed cells further exhibited signs of DNA damage (Figure 1C; Figure S1B), which was unrelated to apoptosis (Figure 1D), or expression of AID or RAG1 (Figure S1C). We next aimed to identify the precise genomic regions that experience DNA damage upon leukemic transformation. To do so, we performed ChIP-seq on apoptotic/dead cell-depleted fractions using antibodies for the DNA-bound DNA damage response protein γH2AX, as done previously by others (Barlow et al., 2013, Rodriguez et al., 2012, Seo et al., 2012). γH2AX regions were identified as described previously (Barlow et al., 2013), with minor modifications to optimize calling of broad γH2AX peaks. γH2AX signals were highly reproducible in a repeat experiment (Figures S1D and S1E). In total, 889 and 960 γH2AX-regions were identified for BCR-ABL1 and C-MYC, respectively (Tables S1 and S2). Interestingly, γH2AX signal intensities at γH2AX-enriched regions were comparable for both BCR-ABL1- and C-MYC-expressing cells (Figure 1E). This may reflect that BCR-ABL1- and C-MYC-induced DNA damage arises in a similar manner (e.g., by replicative stress) or that BCR-ABL1-induced DNA damage mainly arises from activation of BCR-ABL1-induced expression of C-MYC (Sawyers, 1993). We therefore combined both region lists, yielding a merged list of 1,289 γH2AX sites that were used for further comparison (Table S3). γH2AX sites were distributed over all chromosomes (Figure 1F), but the total coverage across chromosomes highly correlated with exon coverage per chromosome (Figure 1G).

### γH2AX-Marked Genomic Regions Occur at B Cell Precursor-Specific Genes and Relate to Endogenous DNA Damage

**Figure 2:**
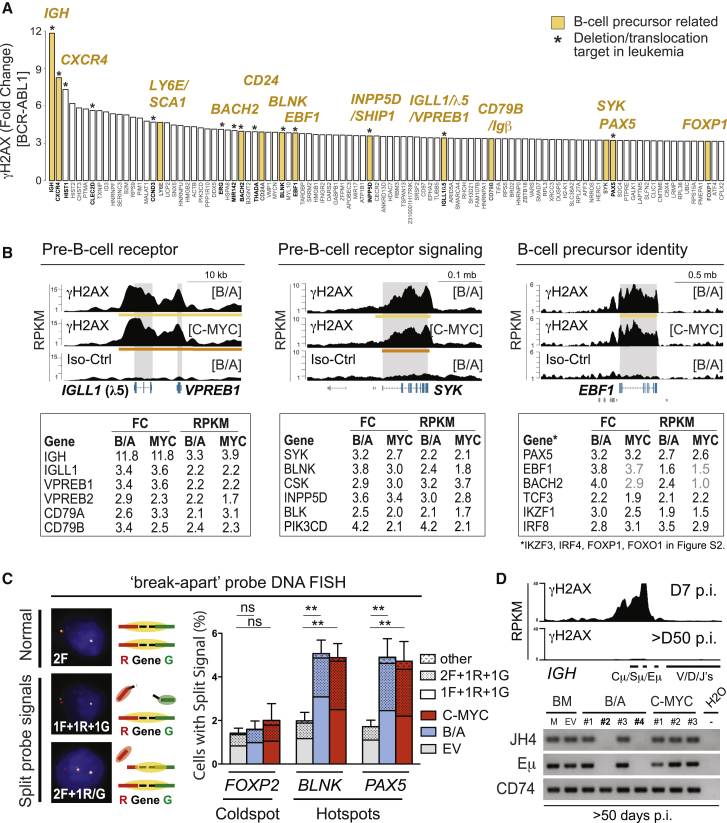
γH2AX-Marked Genomic Regions Occur at B Cell Precursor-Specific Genes and Relate to Endogenous DNA Damage (A) Bar diagram of the top-ranked γH2AX regions identified for BCR-ABL1-transformed B cell precursors. Ranking is based on fold change (FC) of the γH2AX signal compared with control (Iso) signals. Genes that overlap with γH2AX regions are indicated. For multiple regions covering the same gene, regions are combined, and average FC values are shown (i.e., for *HIST1* and *HIST2* genes and for *LY6E*). (B) Top: representative custom track images for genes with γH2AX regions related to the pre-B cell receptor, pre-BCR signaling, and B cell precursor identity. Grey boxes indicate gene body location for the indicated genes. Bottom: tables of γH2AX signal FC values and read density per region expressed as reads per kilobase per million (RPKM) of BCR-ABL1 (B/A) and C-MYC ChIP-seq libraries for genes related to respective gene categories. Values shown in gray did not pass our stringent empirical selection criteria using Statistical Model for Identification of ChIP-Enriched Regions (SICER). Related custom track images can be found in Figures S2A–S2C. (C) Verification of DNA damage at γH2AX hotspots at B lineage genes using DNA FISH and break-apart probes. Left: schematic overview of signals detected. Right: results of five independent experiments for *PAX5* and *BLNK* (γH2AX hotspots) versus *FOXP2* (γH2AX coldspot) (data represent mean ± SEM). (D) Top: custom track visualization of the γH2AX ChIP-seq signal at *IGH* in BCR-ABL1-transformed B cell precursors on day 7 p.i. versus in a BCR-ABL1-induced B cell precursor cell line (>50 days p.i.). Bottom: analysis of the *IGH* locus by PCR using genomic DNA as a template. JH4 proximal intronic and Eμ regions of *IGH* were assessed; CD74 was used as a non-*IGH* control region (one of three repeats is shown). BCR-ABL1-induced cell line #2 is the one analyzed by γH2AX ChIP-seq above.

We next analyzed the identified γH2AX regions for genes that overlap with them. We observed that many of the most prominent regions affected genes specifically expressed by B cell precursors (Figure 2A). Furthermore, most of the crucial genes related to the pre-B cell receptor complex, pre-B cell receptor signaling, and B cell precursor identity-related transcription overlapped with γH2AX-enriched regions present in transformed B cell precursors (Figure 2B; Figures S2A–S2C). Gene ontology (GO) pathway analysis of all γH2AX regions confirmed that they, in many cases, affected immune system-related processes (Figure S2D; Table S4). To verify that identified γH2AX hotspots at B lineage genes correlate to sites of active DNA damage, we next performed DNA fluorescence in situ hybridization (FISH) using “break-apart” probes for two B lineage gene-related γH2AX hotspots (*BLNK* and *PAX5*) and a γH2AX coldspot (*FOXP2*) as a control (Figure 2C). The FISH probes cover the flanking regions of the target genes and are physically separated when a DNA double-strand break (DSB) occurs (split signal) (Figure 2C, left). In agreement with our ChIP-seq results, we could detect a significant increase in split FISH probe signals for the γH2AX hotspots in transformed B cell precursors compared with control cells (Figure 2C, right) but not for the γH2AX-negative region analyzed as a control. Interestingly, when analyzing a successfully transformed B cell precursor line (>50 days after oncogene induction) by γH2AX ChIP-seq, we observed that the most prominent γH2AX region at *IGH* was diminished (Figure 2D, top). To investigate whether this may relate to internal deletions at the *IGH* locus, we analyzed seven successfully transformed B cell precursor cell lines for genomic loss at *IGH* by PCR. We confirmed genomic loss in two of seven cultures, including the cell line initially analyzed by ChIP-seq (Figure 2D, bottom). In summary, our results show that B cell precursor-specific genes represent prominent γH2AX hotspots in transformed B cell precursors, that γH2AX enrichment at B lineage genes correlates to endogenous DNA damage as validated in a limited number of loci by DNA-FISH, and that γH2AX-enriched regions can be subject to genomic deletion during prolonged culture of transformed B cell precursors.

### Identified Regions Locate to Genes Affected by Genomic Lesions in Human B Cell Precursor Leukemia

**Figure 3:**
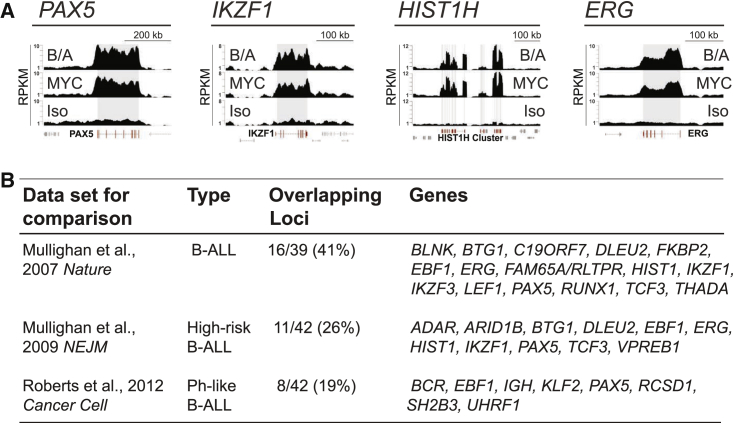
γH2AX Regions Locate to Genes Affected by Genomic Lesions in Human B-ALL (A) Representative custom track images of γH2AX signals at *PAX5*, *IKZF1*, the *HIST1H* gene cluster, and *ERG* for BCR-ABL1 (B/A), C-MYC, and isotype control (Iso) ChIP-seq libraries. Gene models of selected genes are highlighted in red and neighboring genes in gray. Grey boxes indicate gene body location of the indicated gene. (B) Comparison of genes associated with γH2AX-enriched regions in transformed B cell precursors from this study with genes associated with genomic deletions or translocations in human B-ALL. Comparisons were performed against previously published datasets on genomic alterations in human B-ALL, as indicated in the Supplemental Experimental Procedures (Mullighan et al., 2007, Mullighan et al., 2009, Roberts et al., 2012). Only genomic defects were used for comparison that were present in B-ALL, refer to annotated genes, and are present in the mouse genome (explained in the Supplemental Experimental Procedures). The overlapping loci indicated refer to genes that overlap with γH2AX regions identified in BCR-ABL1/C-MYC-transformed murine B cell precursors that were additionally associated with deletions or translocations in human B-ALL identified by the indicated studies. Statistical analysis was done by two-by-two contingency table analysis as by Swaminathan et al. (2015) and confirmed significant enrichment of γH2AX region-associated genes with p < 0.0001, p = 0.002, and p = 0.034 for comparisons with the Mullighan et al., 2007, Mullighan et al., 2009 and Roberts et al. (2012) datasets, respectively. Custom track images for all overlapping genes can be found in Figure S3.

Within the most prominent γH2AX regions, we identified B cell precursor-specific genes but also non-B cell-specific genes known to become defective in leukemia (e.g., *HIST1* and *ERG*) (Figure 3A). To evaluate to what extent genomic alterations in human B-ALL occur at genes that are characterized by γH2AX hotspots in transformed B cell precursors, we compared the list of genes overlapping with γH2AX regions with three datasets documenting gene defects in human leukemia. Only genomic alterations from these datasets that were present in B cell precursor leukemia and that related to genomic deletions or translocations were used for comparison. We found significant enrichment of genes associated with genomic deletions/translocations that similarly overlapped with γH2AX regions identified by us (Figure 3B; Table S5). γH2AX regions also located to genomic loci associated with alterations in B-ALL that were not identified by the above studies (e.g., *BACH2* and *MIR142*). In total, 27 genes reported as defective in human B-ALL were present in our γH2AX region list (Figure S3A) and an additional eight genes related to B-ALL that displayed γH2AX signals but failed to pass our stringent region-calling threshold (Figure S3B).

### γH2AX Hotspots in Transformed B Cell Precursors Occur at Transcriptionally Active Loci

**Figure 4:**
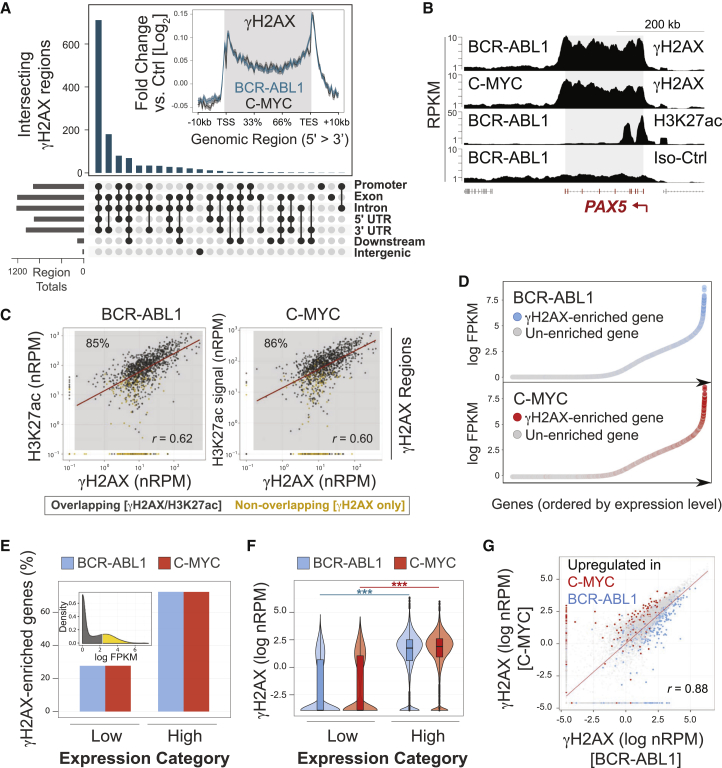
γH2AX Hotspots in Transformed B Cell Precursors Relate to Transcriptionally Active Genes (A) Overlap of γH2AX regions with the indicated gene features for all γH2AX regions identified in transformed B cell precursors. Inset: distribution of γH2AX reads within gene bodies versus upstream and downstream regions for transformed B cell precursors by meta-gene analysis. Signals are normalized to Iso. (B) A representative custom track of γH2AX versus H3K27ac read densities. (C) Scatterplots of γH2AX versus H3K27ac intensities for γH2AX regions. Signals are background-subtracted (“normalized”) RPM values for γH2AX and H3K27ac within identified γH2AX regions from the merged γH2AX region list. For better identification of overlapping regions, regions were extended ± 10 kb as described in the Supplemental Experimental Procedures. Linear regression (red line) and Pearson’s correlation (p < 2.2 × 10^−16^ for both) between γH2AX versus H3K27ac signal intensities is shown for regions that contain both γH2AX and H3K27ac signals (gray shaded area, percentage of total indicated). See Figure S4B for meta-gene analysis of H3K27ac. (D) Relative gene expression levels ranked by fragments per kilobase of transcript per million reads (FPKM) for BCR-ABL1- and C-MYC-transformed B cell precursors. Genes overlapping γH2AX-enriched regions are highlighted as indicated. (E and F) Genes were categorized as high- or low-expressed relative to the median expression level across the population of all expressed genes. Inset, left: kernel density plot of FPKM values; colors indicate expression categories. Relative distribution of γH2AX-enriched genes between expression categories (E) and distribution of normalized γH2AX values measured for extended gene regions between expression categories (F) are shown. The shaded area indicates kernel density overlaid with a standard boxplot, and stars indicate the highly significant difference between categories (p < 1 × 10^−15^; Mann-Whitney *U* test). (G) Scatterplot of the difference in γH2AX signal (nRPM) across extended gene regions between BCR-ABL1- and C-MYC-transformed B cell precursors. Differentially expressed genes are highlighted in blue or red as indicated. Linear regression (red line) and Pearson’s rho (*r*) are indicated.

We next analyzed the genomic location of the identified regions in more detail. 788 of 1,289 γH2AX regions overlapped with multiple genes, leading to a total overlap of 1,289 regions with 2,300 genes. γH2AX regions often specifically covered the gene body of a respective gene, which was confirmed by meta-gene analysis (Figure 4A). In agreement with gene body-associated fragility in transformed B cell precursors, cross-analysis of structural variations (SVs) previously identified in human leukemia also showed breakpoints to be enriched within gene bodies (Figure S4A). To assess whether γH2AX region formation relates to transcription, we next generated ChIP-seq libraries for the chromatin mark H3K27ac, which is present at transcriptionally active promoters and enhancers (Shlyueva et al., 2014). Analysis of H3K27ac distribution confirmed accumulation at transcription start sites (TSSs) in transformed B cell precursors (Figure S4B). Furthermore, most γH2AX regions exhibited H3K27ac at respective promoters, and abundance of these two histone marks at overlapping regions showed significant correlation (Figures 4B and 4C). Analysis by RNA sequencing (RNA-seq) confirmed γH2AX regions to be enriched among highly expressed genes (Figures 4D and 4E) and that highly expressed genes exhibit greater γH2AX intensities (Figure 4F). Furthermore, genes differentially expressed between BCR-ABL1 and C-MYC conditions displayed a concomitant change in γH2AX signal intensity (Figure 4G). In agreement with increased γH2AX signals at highly transcribed loci in transformed B cell precursors, we also observed that induced expression of a transgenic fluorochrome (DS-RED) in transformed cells resulted in elevated γH2AX accumulation at the related expression cassette (Figure S4C).

### Analysis of Convergent and Divergent Transcription, R Loop-Forming Sequences, and Early-Replicating Fragile Sites at γH2AX Regions

**Figure 5:**
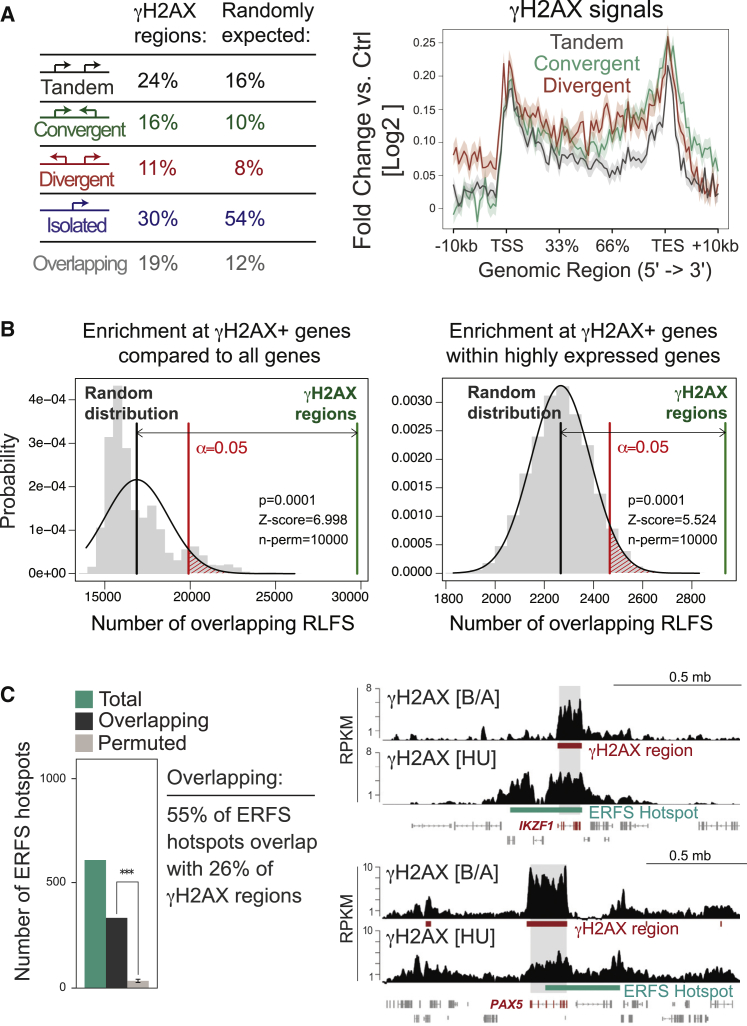
Analysis of Convergent and Divergent Transcription, R Loop-Forming Sequences, and Early-Replicating Fragile Sites at γH2AX Regions (A) Left: gene pairs within 10 kb were categorized as tandem, convergently, or divergently transcribed, as indicated in the respective diagrams, or as isolated (no additional gene within 10 kb) or overlapping. The proportion of genes in each subset is indicated for both γH2AX region-associated genes (left) and the distribution across the whole genome (right). Right: average fold change in γH2AX signal in BCR-ABL1 versus isotype control across gene bodies for each transcription orientation category, normalized for gene lengths (p < 1 × 10^−6^ for both convergent versus tandem and divergent versus tandem comparisons using Mann-Whitney test). (B) Analysis for the enrichment of predicted RLFSs at γH2AX-associated genes. The total count of RLFSs overlapping γH2AX-associated genes (green line) was compared with the permuted null distribution of total RLFSs (gray bars) measured by random sampling of the gene population (the black line indicates mean, and red indicates a significance threshold of 0.05). Significant enrichment of RLFSs at γH2AX-associated genes is observed both among all genes (left) and when analysis is restricted to only the 1,000 most highly expressed genes (right). (C) Left: bar diagram showing the number of ERFS hotspots (Total) versus the number of these sites that overlap with γH2AX regions in transformed B cell precursors (Overlapping). The significance is indicated by comparison with the mean permuted number of overlaps from random re-sampling of equivalently sized regions within the genome. Right: HU-induced versus BCR-ABL1-induced γH2AX signal patterns at two representative loci by custom track visualization. The bars indicate the ERFS hotspots (mint) and BCR-ABL1-induced γH2AX regions (red). Grey boxes indicate gene body location of the highlighted gene.

To understand the origins of transcription-induced γH2AX regions in transformed B cell precursors, we analyzed whether γH2AX regions are associated with known causes of transcription-related fragility. We first analyzed γH2AX regions for their relation to convergent and divergent transcription, which have been associated with genomic instability (Barlow et al., 2013, Heinäniemi et al., 2016, Pannunzio and Lieber, 2016). γH2AX regions were enriched at gene pairs compared with isolated genes but not further enriched for convergent or divergent gene orientation compared with tandem orientation (Figure 5A, left). However, in agreement with increased fragility at regions with convergent and divergent transcription, we observed that γH2AX signals were elevated at convergent/divergent gene pairs compared with tandem genes (Figure 5A, right; p < 1 × 10^−6^ for both, convergent versus tandem and divergent versus tandem comparisons; Mann-Whitney test). We next assessed whether γH2AX regions may relate to R loop formation, which can promote fragility by exposure of single-stranded DNA (ssDNA) or interference with replication. To do so, we predicted R loop-forming sequences (RLFSs) for the mouse genome as described previously (Jenjaroenpun et al., 2015) and analyzed γH2AX regions for enrichment of RLFSs. Of note, RLFSs do not indicate the actual presence of R-loops but an increased propensity of a nucleotide sequence to promote R-loop formation. Likewise, detection of RNA-DNA hybrids by DNA:RNA immunoprecipitation sequencing (DRIP-seq) analysis has shown significant enrichment of R-loops at computationally predicted RLFSs (Heinäniemi et al., 2016). We found that genes marked by γH2AX were enriched for RLFSs compared with the gene population as a whole (Figure 5B, left) or when considering only highly expressed genes (Figure 5B, right), suggesting an increased likelihood of R loop formation at γH2AX regions. We next compared γH2AX regions to locations of early replication fragile sites (ERFSs) (Barlow et al., 2013), which accumulate DNA DSBs and γH2AX in response to hydroxyurea (HU)-induced replicative stress. ERFS hotspots significantly overlapped with the γH2AX regions identified by us (55% of ERFSs overlapped with 26% of γH2AX regions) (Figure 5C, left); however, HU-induced γH2AX signals at ERFSs differed from the signals present in transformed B cell precursors at overlapping regions (Figure 5C, right).

### Lineage-Specific Transcription Predisposes Lineage-Specific Genes to DNA Damage

**Figure 6:**
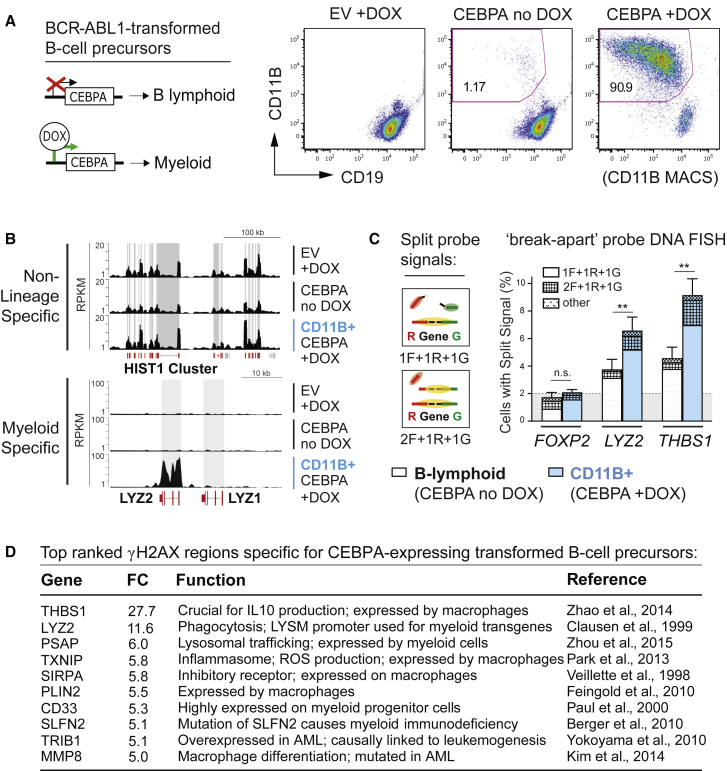
Induction of a Myeloid Lineage Phenotype in Transformed B Cell Precursors Causes DNA Damage at Myeloid-Specific Genes (A) Left: Schematic of the experimental strategy for induction of a myeloid phenotype in transformed B cell precursors using DOX-inducible CEBPA expression. Right: DOX/CEBPA-mediated induction of the myeloid phenotype verified by flow cytometry using CD11B as a myeloid lineage and CD19 as a B lymphoid lineage marker. EV-transduced lymphoid leukemia cells were used as a control. Anti-CD11B magnetic bead based cell sorting (MACS) was performed on DOX-treated cells with inducible CEBPA to reach a purity of >90% CD11B-positive cells for ChIP-seq analysis. (B) Custom track visualization of γH2AX read densities for non-lineage-specific (*HIST1* cluster, top) and myeloid lineage-specific gene loci (*LYZ2*, bottom). Grey boxes indicate gene-body location of the indicated gene. (C) Assessment of myeloid γH2AX ChIP-seq hotspots identified in CEBPA + DOX cells using DNA FISH and break-apart probes as described in Figure 2C. Results of five independent experiments for *LYZ2* and *THBS1* (myeloid γH2AX hotspots) versus *FOXP2* (coldspot) are shown (data represent mean ± SEM). (D) Table of the ten top-ranked genes with significant γH2AX signals specific for CEBPA + DOX cells (by fold change). Full references can be found in the Supplemental Information.

To functionally investigate the potential link between lineage-specific transcription and DNA damage at lineage-specific genes, we next investigated whether a fundamental change in the cell-type-specific transcriptional program of transformed B cell precursors may cause a concomitant change in DNA damage hotspot localizations at lineage-specific genes. To do so, we used a similar model as before, in which B cell precursors are transformed by the BCR-ABL1 oncogene but with the addition of an inducible CEBPA transgene, which promotes myeloid-specific transcription and a corresponding myeloid lineage phenotype in B cell precursors (Chen et al., 2015; Figure 6A). This allowed us to examine de novo DNA damage-sensitive sites in transformed B cell precursors by γH2AX ChIP-seq that occur as a consequence of myeloid lineage-specific transcription. Indeed, induction of a myeloid phenotype (CEBPA + doxycycline [DOX]) was associated with de novo γH2AX signals at myeloid-specific genes that were not present in our lymphoid lineage controls (Figure 6B, bottom; Table S6). γH2AX signals at non-lineage-specific genes showed no difference between lymphoid and myeloid phenotypes (Figure 6B, top). γH2AX signals at B cell-specific genes only showed a minor reduction in CEBPA-expressing cells compared with controls, possibly reflecting an incomplete loss of the B cell phenotype in our experiments. To confirm DNA damage at myeloid γH2AX hotspots, we performed DNA FISH as described in Figure 2C but using probes for two myeloid hotspots (*LYZ2* and *THBS1*). In agreement with our ChIP-seq results, we detected increased split probe signals for *LYZ2* and *THBS1* by DNA FISH in the myeloid compared with the lymphoid fraction (Figure 6C). Analysis of the top-ranked γH2AX ChIP-seq regions specific for cells with the myeloid phenotype further showed that all of them related to myeloid lineage or function (Figure 6D). GO pathway analysis additionally confirmed high enrichment of myeloid-specific genes overlapping with γH2AX regions identified in cells with the myeloid phenotype (Table S7). These findings provide a functional validation of our hypothesis that lineage-specific transcriptional programs promote fragility of lineage-specific genes in transformed B cell precursors.

## Discussion

Oncogenic lesions in hematopoietic progenitor cells give rise to B lymphoid and myeloid malignancies that, at the time of diagnosis, often carry secondary genomic defects that contribute to the disease. The most prominent defects in lymphoid leukemia genomes affect lineage-specific genes (Mullighan et al., 2007, Mullighan et al., 2009, Zhang et al., 2011) that guide respective hematopoietic differentiation and function. Defects at lineage-specific genes are likely to contribute to the differentiation arrest present in leukemia cells, which is thought to represent a causative event for malignant transformation (Rosenbauer and Tenen, 2007). It is unclear, however, whether defects at lineage-specific genes arise from random mutation and selection or whether lineage-specific genes are more likely to become defective. Although our experiments focus on B cell precursors, our results argue for the latter. During malignant transformation of B cell precursors by the leukemia-inducing oncogenes C-MYC and BCR-ABL1, B lineage-specific genes become prominent γH2AX hotspots that relate to DNA DSBs, as shown by DNA FISH for *PAX5* and *BLNK*. γH2AX accumulation is associated with increased transcription, and induction of myeloid-specific transcription in transformed B cell precursors by CEBPA-mediated reprogramming further causes de novo γH2AX hotspots at myeloid-specific genes that relate to DNA DSBs, as shown by DNA FISH for *LYZ2* and *THBS1*. As we assessed only a relatively small number of γH2AX hotspots by DNA FISH, further genome-wide investigation using other tools than ChIP-seq, such as translocation capture sequencing (TC-seq) (Klein et al., 2011), DSB-Capture (Lensing et al., 2016), or DSB end sequencing (END-seq) (Canela et al., 2016) will be beneficial to corroborate DNA DSBs at all identified γH2AX hotspots. Such analyses would also allow a more detailed characterization of the precise breakage sites within identified γH2AX hotspots because γH2AX spreading can occur over extended distances around a DNA DSB (e.g., as shown by Bothmer et al., 2011). Nevertheless, our results show that lineage-specific transcription and the associated fragility of highly transcribed loci represents a major cause of instability at lineage genes in transformed B cell precursors. Such predisposition to DNA damage is likely to increase the occurrence of genomic alterations at B lineage-specific genes and to facilitate subsequent positive selection during clonal evolution as observed in human B-ALL. Notably, with the two experimental strategies we performed (oncogene induction in primary B cell precursors and CEBPA-induced reprogramming in BCR-ABL1-transformed B cell precursors), we analyzed DNA damage at an early stage of malignant transformation as well as at a late stage (primary B cell precursors were analyzed 7 days after oncogene induction [p.i.], whereas CEBPA-reprogrammed cells were analyzed >30 days p.i.). Because we observed fragility of lineage-specific genes in both models, our experiments indicate that this instability phenotype is not limited to the acute response to oncogene activation but represents a characteristic of both early- and long-term-transformed cells.

Although our results provide a link between the high frequency of genomic defects of lineage genes in hematopoietic cancer and transcription-related DNA damage observed in other experimental contexts, transcription can cause genomic fragility for many reasons. For example, transcription exposes ssDNA, which is more fragile and vulnerable to DNA damage. ssDNA can be formed at sites of premature transcription termination (Wang et al., 2014) or within R-loops that occur naturally during transcription (Santos-Pereira and Aguilera, 2015, Skourti-Stathaki and Proudfoot, 2014). R-loops have been shown to specifically associate with highly expressed genes in yeast (Chan et al., 2014), and absence of the DNA damage response protein BRCA1 has been shown to promote R loop-dependent DNA damage at transcriptional pause sites (Hatchi et al., 2015). R loop-associated DNA damage further causes γH2AX accumulation (Hatchi et al., 2015), and RLFSs have been recently described at structural variation hotspots (Heinäniemi et al., 2016). Similarly, we observed that transcription-associated γH2AX hotspots in transformed B cell precursors are strongly enriched for RLFSs. Although RLFSs do not indicate the actual presence of R-loops but an increased likelihood of R loop formation, RLFSs do have significant predictive power for R-loops (Heinäniemi et al., 2016, Jenjaroenpun et al., 2015). However, RLFSs are abundantly encoded throughout the genome within and outside of γH2AX regions (Figure S4D), but cell-type-specific transcription programs are likely to dictate which of these loci actively form R-loops and, consequently, which genomic regions exhibit fragility. In agreement with this, we showed that γH2AX hotspot formation and related DNA DSB induction can be altered by changing lineage-specific transcription.

Additional factors may further amplify transcription-induced fragility during leukemic transformation, such as divergent and/or convergent transcription, which, similarly, can lead to exposure of ssDNA and which have been associated with an increased risk of genomic instability (Barlow et al., 2013, Heinäniemi et al., 2016, Pannunzio and Lieber, 2016). Indeed, we observed elevated γH2AX signals at convergently and divergently transcribed genes. Alternatively, transcription-related DNA damage might also occur as a result of Topoisomerase 2 (TOP2)-mediated DNA DSB induction, which has been described to occur during rapid transcriptional activation of induced genes (Bunch et al., 2015, Madabhushi et al., 2015). Notably, γH2AX signals reported for TOP2-induced DNA damage exhibited a gene body-restricted pattern similar to the pattern present in transformed B cell precursors (Bunch et al., 2015, Madabhushi et al., 2015). However, we were unable to assess whether TOP2-related DNA damage may additionally promote γH2AX hotspot formation and consecutive DNA damage in our experimental model because of immediate induction of apoptosis in response to TOP2 inhibition (data not shown).

Besides transcription itself, interference between transcription and replication has been repeatedly reported as a potential reason for DNA damage (e.g., by Barlow et al., 2013, and Jones et al., 2013). For example fragile sites, which are especially prone to DNA damage during replicative stress, often coincide with sites of high transcription (common fragile sites [CFSs] often coincide with large and transcriptionally active genes [Helmrich et al., 2006], and ERFSs, similarly, associate with transcriptionally active DNA [Barlow et al., 2013]). In agreement with potential transcription-replication conflicts in transformed B cell precursors, we observed an overlap of 55% of reported ERFS hotspots (Barlow et al., 2013), with 26% of the γH2AX regions identified here in transformed B cell precursors. Similarly, a recent report linked replicative stress in RAS-expressing immortalized fibroblasts to transcription-replication conflicts (Kotsantis et al., 2016). However, RAS-induced transcriptional interference with replication in fibroblasts related to upregulation of the TATA box binding protein TBP, but we did not observe any increase of TBP expression in transformed B cell precursors by RNA-seq (data not shown). This is in agreement with several studies by others that showed that oncogenic stress and related DNA damage can significantly differ between cell types and individual oncogenes (Maya-Mendoza et al., 2015, Miron et al., 2015). Nevertheless, we observed that ∼85% of the γH2AX regions identified here in transformed B cell precursors overlapped with early replication domains described for the murine B cell lymphoma line CH12 (1,091 of 1,289 regions, p < 1 × 10^−4^; permutation test) (Stamatoyannopoulos et al., 2012), suggesting interference of transcription with replication as a potential cause for amplification of transcription-induced fragility in transformed B cell precursors. However, because replication timing is highly correlated with transcriptional activity in higher eukaryotes (Schübeler et al., 2002), the relative importance of replication interference in transformed B cell precursors compared with the direct effects of transcription alone remains to be determined.

## Experimental Procedures

### Mice

For experiments on early-transformed B cell precursors, *53BP1*^−/−^ mice (Ward et al., 2003) were used. For experiments involving CEBPA expression (Figure 6), BCR-ABL1-transformed B cell precursors from wild-type mice were used. *53BP1*^−/−^ mice were a kind gift from Dr. Simon Boulton (The Francis Crick Institute). All experiments were performed in agreement with the Animals (Scientific Procedures) Act 1986 (ASPA) guidelines and regulations and protocols approved by the Home Office.

### Cell Culture

Bone marrow (BM) was isolated from femora and tibiae of mice and depleted of erythrocytes using ammonium-chloride-potassium (ACK) lysis buffer (Gibco). Primary B cell precursors were then enriched and maintained by ex vivo culture in Iscove’s Modified Dulbecco’s Medium (IMDM) containing 20% fetal calf serum (FCS) (Invitrogen), 1% penicillin/streptomycin (Gibco), 1% L-Glutamine (Gibco), and 50 μM 2-mercaptoethanol (Sigma) at 37°C in a humidified incubator with 5% CO_2_ in the presence of 10 ng/mL of recombinant mouse interleukin-7 (IL-7) (PeproTech). DOX-inducible CEBPA and control cells were those reported by Chen et al. (2015) and were kept in complete IMDM as described above but without addition of IL-7. Selection of expression cassettes was maintained using 2 μg/mL Puromycin (Sigma) and 500 μg/mL Geneticin/G418 (Sigma). CEBPA-mediated reprogramming was induced by addition of 1 μg/mL DOX (Sigma) for 7 days. Splenic B cells used as controls for western blot and qRT-PCR were isolated from wild-type mice as described previously (Feldhahn et al., 2012).

### Viral Transduction

Transduction of B cell precursors was performed as described previously (Feldhahn et al., 2012) but using X-tremeGENE 9 DNA transfection reagent (Roche) and MIGRI vectors. Empty MIGR1 vector and human BCR-ABL1^P210^-MIGR1 were kind gifts from Danilo Perrotti (University of Maryland School of Medicine). Mouse C-MYC was sub-cloned into MIGR1 from C-MYC-MIG using XhoI (New England Biolabs). Transduction with MIGRI vectors was performed by spinoculation on days 2 and 3 after start of the cell culture. Details on vectors, transduction, and experimental conditions for experiments involving inducible *DS-RED* expression can be found in the Supplemental Experimental Procedures.

### ChIP-Seq, ChIP-qPCR and RNA-Seq

Cells were prepared for ChIP-seq or ChIP-qPCR analysis according to Yamane et al. (2013) with minor modifications. For each experiment, harvested cells were depleted of dead/apoptotic cells using the dead cell removal kit (Miltenyi Biotec) before further processing. For CEBPA experiments, myeloid cells were additionally enriched using anti-CD11B micro-beads (Miltenyi). Primary transformed and untransformed B cell precursors were typically harvested on day 7 after transduction; for CEBPA experiments, cells were harvested after 7 days of DOX addition. For library preparation, the NEBNext ChIP-Seq Library Prep Master Mix Set and NEBNext Multiplex Oligos for Illumina (NEB) were used. For RNA-seq, cells were isolated, cultured, and harvested as for ChIP-seq experiments. RNA was extracted in duplicate experiments from 5 × 10^6^ cells using the QIAGEN RNeasy mini kit. Libraries were generated from pre-enriched mRNA using the NEBNext mRNA Library PrepReagent Set for Illumina (NEB) and pre-amplified using the NEBNext Multiplex Oligos for Illumina (NEB). A detailed description of the experimental procedures, bioinformatic analyses, and statistics is available in the Supplemental Experimental Procedures.

### Statistical Analysis

Statistical analysis of DNA FISH and qRT-PCR data was done by paired Student’s t test ± SEM using GraphPad Prism software. For comparison of γH2AX regions to published datasets on gene defects in B-ALL, two-by-two contingency table analysis was performed as by Swaminathan et al. (2015). For statistical analyses of ChIP-seq and RNA-seq data, please refer to the Supplemental Experimental Procedures.

### DNA FISH

Cultured cells were depleted of dead/apoptotic cells using the dead cell removal kit (Miltenyi). 2–5 × 10^6^ cells were then cultured in fresh medium with 100 ng/mL Colcemid (Gibco) for 4 hr and processed as described previously (Barch et al., 1997). DNA FISH was performed using custom dual-color break-apart probes (Agilent Technologies) diluted 1:100 in ULTRAhyb (Ambion) and hybridized overnight. Automated FISH capture and semi-automated analysis were performed using CytoVision GSL-120 (Leica). Detailed information on DNA FISH probes will be provided upon request.

### qRT-PCR and PCR

For qRT-PCR on IL-7-cultured B cell precursors, cells were typically harvested on day 7 after transduction, dead/apoptotic cell-depleted, and snap-frozen on dry ice. For thymus and BM, cells were isolated freshly from *53BP1*^−/−^ mice and snap-frozen on dry ice. Stimulated B cells were obtained from the spleen and cultured as described previously (Feldhahn et al., 2012). RNA was isolated as for RNA-seq, and cDNA was generated using the RevertAid cDNA synthesis kit (Thermo Fisher Scientific). qPCR was performed using a SYBR green master mix (Sigma JumpStart) on a StepOnePlus platform (Thermo Fisher Scientific). Information of primers, PCR conditions, and analysis can be found in the Supplemental Experimental Procedures. Analysis of the *IGH* locus for genomic loss was done as by Jankovic et al. (2013) using the primers and conditions described.

### Flow Cytometry

Survival indicated by forward scatter (FSC)/side scatter (SSC) as well as GFP or DS-RED expression of transduced cells was assessed by flow cytometry using FACSCalibur or LSRFortessa (BD Biosciences). For a more detailed analysis of viability and apoptosis induction, the Annexin V-PE (phycoerythrin) apoptosis detection kit (BD Biosciences) in combination with 7-AAD (BD Biosciences) was used. For cell surface staining of single-cell suspensions, the following anti-mouse antibodies were used: CD16/32 (Fc Block, clone 2.4G2, BD Biosciences) and CD19-APC (eBio103), B220-PerCPCy5.5 (RA3-6B2), IgM-APCe780 (II/41), CD43-PE (eBioR2/60), CD24-FITC (30-F1), and CD11b-PECy7 (M1/70) (eBioscience).

### Western Blot

Protein lysates were made from snap-frozen cell pellets using radioimmunoprecipitation assay (RIPA) buffer (50 mM Tris-HCl [pH 8.0], 0.5 mM EDTA, 0.1% SDS, 1% NP-40/IGEPAL (octylphenoxypolyethoxyethanol), 0.5% sodium deoxycholate, and 150 mM NaCl). 10–20 μg of proteins were analyzed by SDS-PAGE gel electrophoresis and western blot using the Mini-PROTEAN blotting module and Tris-Glycine eXtended (TGX) stain-free pre-cast gels (Bio-Rad). For detection of specific proteins, the following antibodies were used: anti-phospho-histone H2A.X (serine 139, γH2AX) clone JBW301 (Millipore), anti-phospho-P53 (serine 15, Cell Signaling Technology), anti-α-tubulin (Abcam), anti-LAMIN B1 (Abcam), and anti-AID (L7E7) (Cell Signal).

## Author Contributions

B.B. performed the cell culture, ChIP-seq, qRT-PCR, and western blot. M.E.R. analyzed all deep sequencing data. P.C.M. performed RNA-seq, DNA FISH, and ChIP qPCR. L.C. helped with RNA-seq processing. J.T. performed the DS-RED experiment. K.B. optimized γH2AX region calling. A.R. supervised DNA FISH. M.M., J.F.A., and J.S. contributed by helpful discussion. N.F. designed and supervised the study, wrote the manuscript, and performed some experimental work.

## Accession Numbers

The accession number for the RNA-seq and ChIP-seq data reported in this paper is NCBI GEO: GSE80682.

## Supplemental Information

Document S1. Supplemental Experimental Procedures and Figures S1–S4

Table S1. BCR-ABL1 γH2AX Regions Summary, Related to Figures 1 and 2

Table S2. C-MYC γH2AX Regions Summary, Related to Figures 1 and 2

Table S3. Merged γH2AX Regions Summary, Related to Figures 1, 4, and 5

Table S4. Merged γH2AX Regions GO Results, Related to Figure S2D

Table S5. Gene Defects in Human B-ALL versus γH2AX Regions, Related to Figure 3

Table S6. CEBPA + DOX-Specific γH2AX Regions Summary, Related to Figure 6

Table S7. CEBPA + DOX-Specific γH2AX Regions GO Results, Related to Figure 6

Document S2. Article plus Supplemental Information
