# Supplementary material for: Lineage-Specific Genes Are Prominent DNA Damage Hotspots during Leukemic Transformation of B Cell Precursors
Source: Cell Rep. 2017 Feb 14;18(7):1687–98. doi: 10.1016/j.celrep.2017.01.057 (PMC5318656; doi:10.1016/j.celrep.2017.01.057)
Supplement: Document S1. Supplemental Experimental Procedures and Figures S1–S4 [file mmc1.pdf]

Cell Reports, Volume *18*

Supplemental Information

Lineage-Specific Genes Are Prominent DNA Damage Hotspots during Leukemic Transformation of B Cell Precursors

Bryant Boulianne, Mark E. Robinson, Philippa C. May, Leandro Castellano, Kevin Blighe, Jennifer Thomas, Alistair Reid, Markus Müschen, Jane F. Apperley, Justin Stebbing, and Niklas Feldhahn

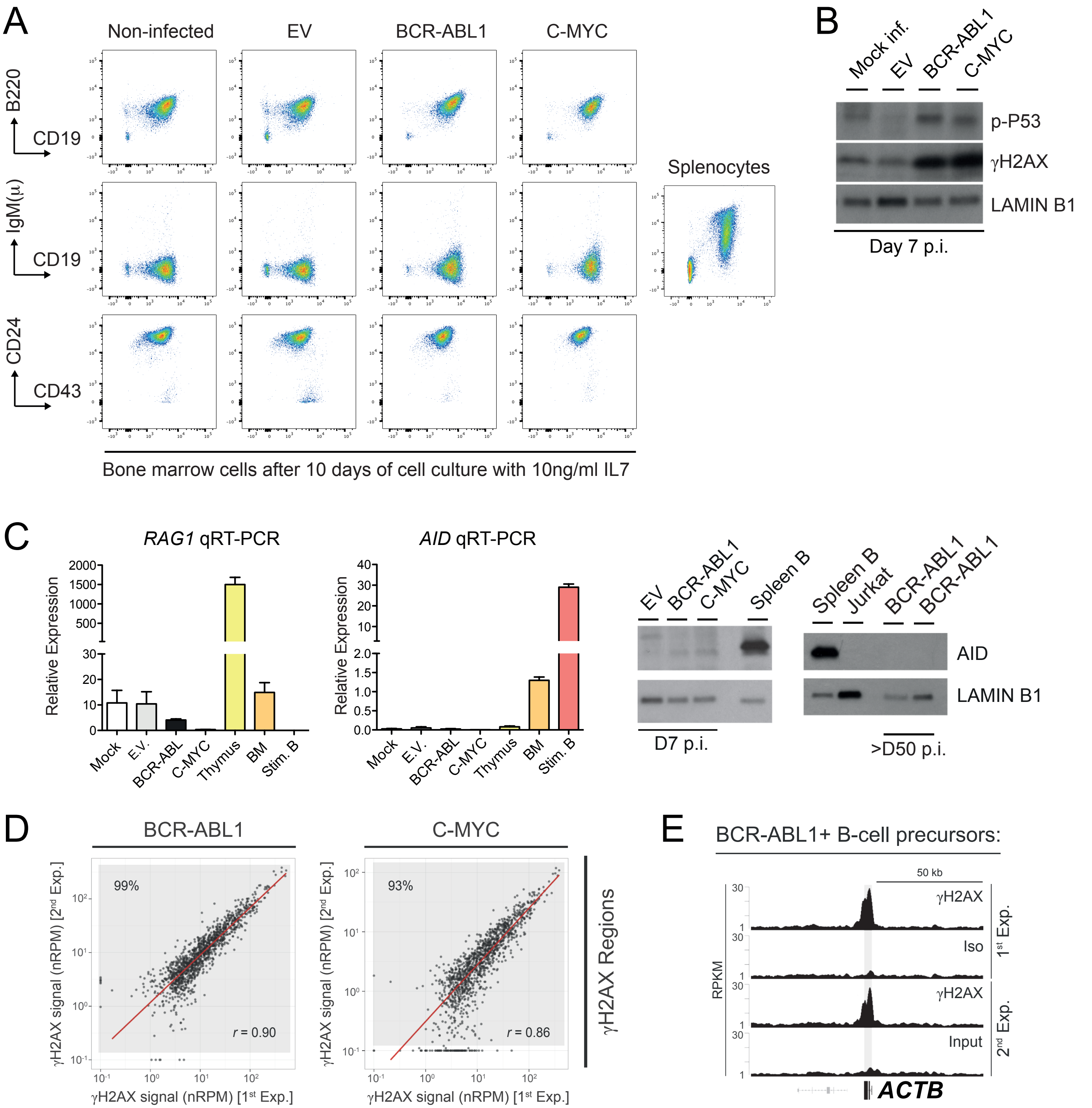

Figure S1. Analysis of DNA damage in transformed B-cell precursors by γH2AX-ChIP-Seq, Related to Figure 1
A) Analysis of B-cell precursor enrichment after 10 days of cell culture of total bone marrow in presence of IL7 by flow cytometry using antibodies indicated. BCR-ABL1, C-MYC and EV (empty vector) cells were additionally transduced on day 2 and 3 by respective MIGRI encoding retrovirus. As a positive control for functionality of the anti-IgM antibody a staining of splenocytes is shown. B) Western blot analysis of infected vs. non-infected (mock) B-cell precursor cells for DNA damage marks γH2AX and p-P53^{Ser15} using LAMIN B1 as loading control (independent experiment to Figure 1C/D). C) qRT-PCR analysis for *RAG1* (left) and *AID* (middle) expression in transformed vs. untransformed B-cell precursors (D7 post oncogene induction [p.i.]) using total thymus and splenic LPS/IL4-stimulated mature B-cells as positive controls for RAG1 and AID, respectively. Data represents three independent experiments, error bars indicate mean +/- SEM. (Right) Analysis of AID protein abundance in transformed B-cell precursors by Western blot using the antibodies indicated. D) Comparison of γH2AX-ChIP-seq repeat experiments for BCR-ABL1 and MYC transduced B-cell precursors by scatter plot visualization. Normalized RPM (nRPM) values of the γH2AX signal within identified γH2AX regions (merged list) are shown for BCR-ABL1 (left) and MYC (right), red line represents linear regression fit. E) Comparison of γH2AX-ChIP-seq repeat-experiments by custom track visualization. A representative image for BCR-ABL1-transduced B-cell precursors is shown. The isotype control library (Iso) visualizes the background ChIP-seq signal. Gene bodies and orientation is indicated with black bars.

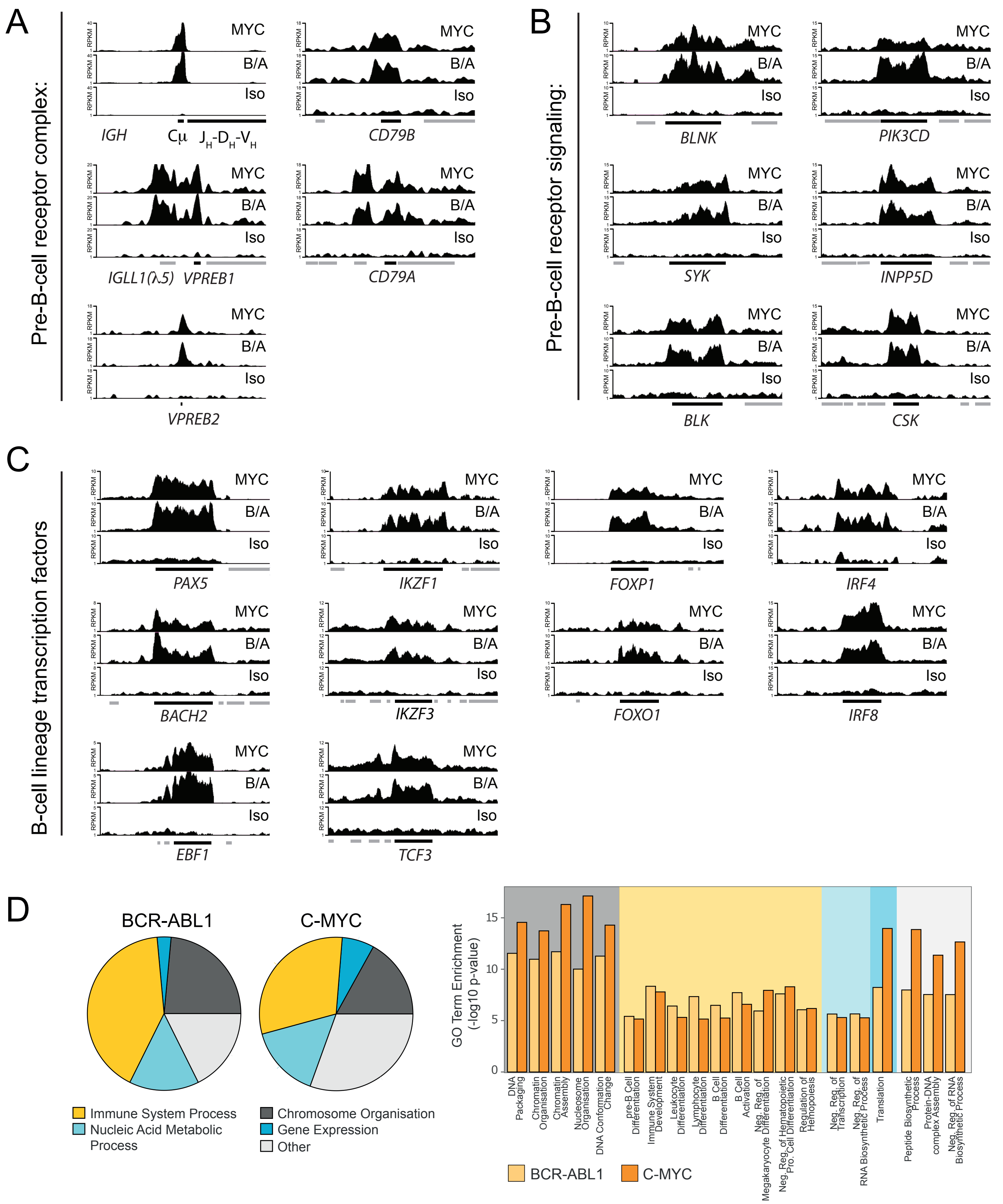

Figure S2. Enrichment of the DNA damage response protein γH2AX at B-cell lineage genes, Related to Figure 2
A-C) γH2AX-ChIP-seq custom track images of B-cell precursor cells expressing C-MYC or BCR-ABL1 (B/A) for B-cell lineage genes. These include (A) genes forming the pre-B-cell receptor complex, (B) genes required for pre-B-cell receptor signal transduction, and (C) genes required for B-cell lineage commitment/differentiation. The isotype control library (Iso) visualizes the background ChIP-seq signal. Gene bodies of the related gene are indicated with black bars, neighboring genes in grey. D) Gene ontology (GO) pathway enrichment analysis of identified γH2AX regions: (Left) Pie charts showing GO enrichment categories. (Right) Bar diagram showing in more detail the GO pathways that resemble enrichment categories of Pie charts on the left.

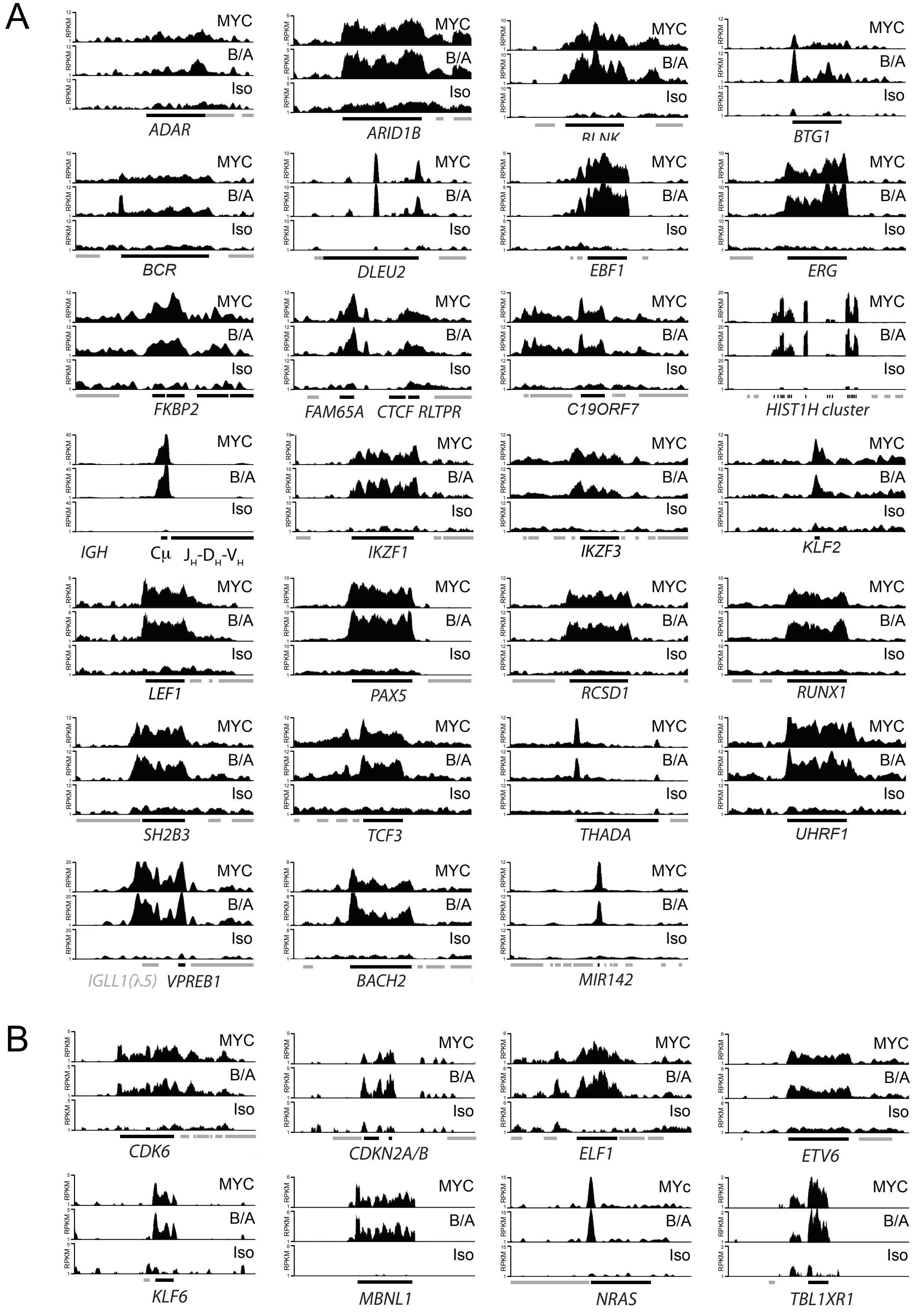

Figure S3. Enrichment of γH2AX at genes known to become defective in B-ALL, Related to Figure 3
A) γH2AX-ChIP-seq custom track images of B-cell precursor cells expressing C-MYC or BCR-ABL1 (B/A) for 27 genes with significant γH2AX signals identified by SICER and that are reported to become defective in human B-ALL. The isotype control library (Iso) visualizes the background ChIP-seq signal. Gene bodies of related genes are indicated with black bars, neighboring genes in grey. B) Visualization as in (A) for 8 genes reported to become defective in B-ALL that exhibit γH2AX signals but did not pass our stringent selection criteria by SICER.

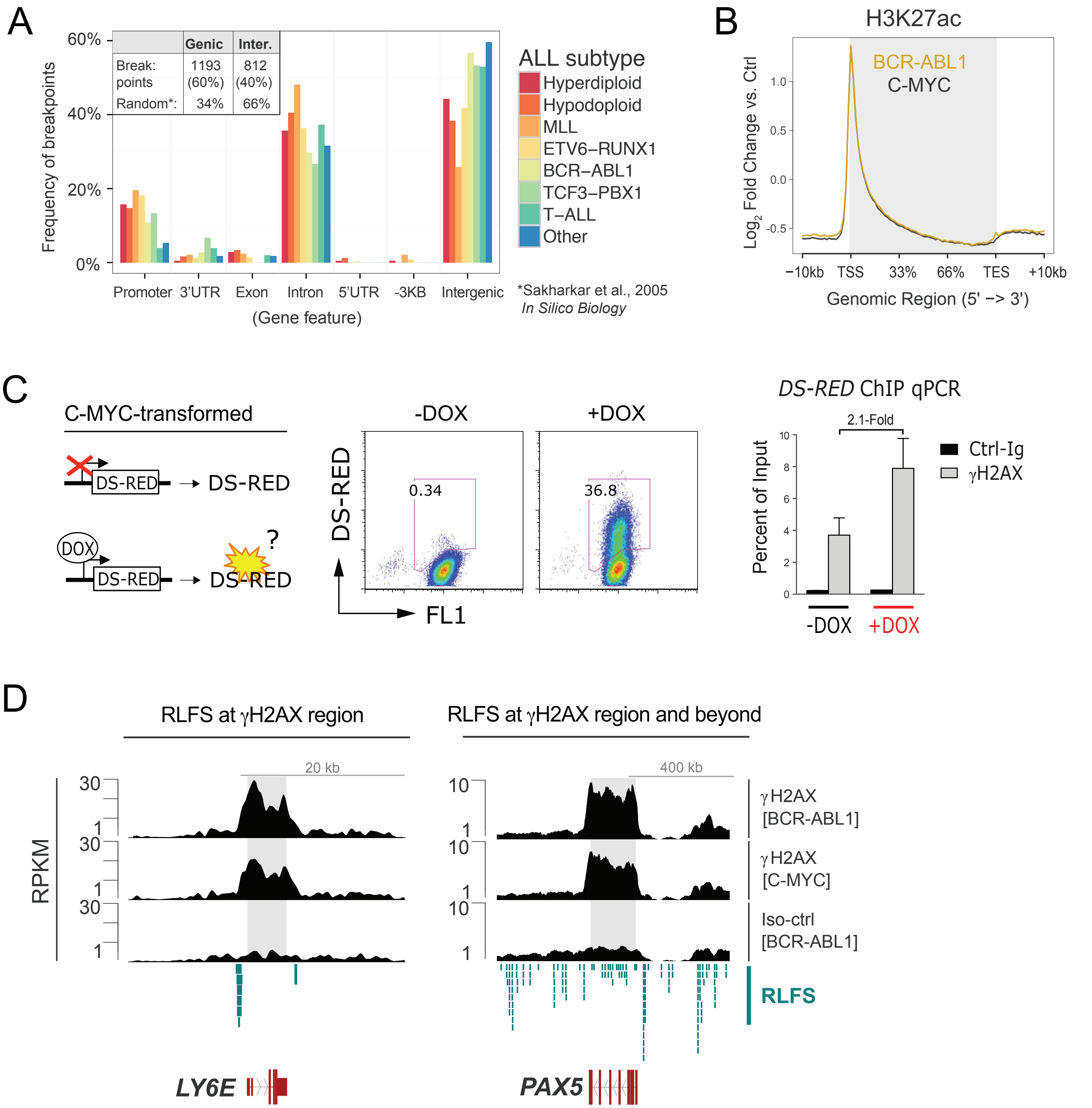

Figure S4. H3K27ac and γH2AX in transformed B-cell precursors, Related to Figure 4 and 5
A) Analysis of structural variation (SV) breakpoint (Bp) frequencies from SVs reported for human leukemia genomes for their distribution over genic and intergenic regions as indicated. Inset shows the sum of Bp's for all data sets analyzed compared to the normal distribution of genic and intergenic DNA within the human genome. B) Distribution of H3K27ac reads within gene bodies vs. upstream and downstream regions for transformed B-cell precursors by meta-gene analysis. Signals are normalized to isotype-Ctrl ChIP-seq library (Iso). C) Analysis of γH2AX accumulation upon Doxycycline (DOX)-induced *DS-RED* expression in C-MYC-transformed B-cell precursors by ChIP qPCR. (Left) Schematic diagram of experimental strategy for the DS-RED experiment. (Middle) Verification of DOX-induced *DS-RED* expression in C-MYC-transformed B-cell precursors by flow cytometry. (Right) Bar diagram showing the increase of *DS-RED* sequence within the γH2AX-ChIP fraction normalized to input DNA used for ChIP, for +DOX vs. -DOX treated cells, as determined by ChIP qPCR. D) Custom track images of the γH2AX-ChIP-seq signal at two genes with γH2AX regions in BCR-ABL1- and C-MYC-transformed B-cell precursors and respective locations of predicted R-loop forming sequences (RLFS) (mint).

Supplemental Table Legends

Table S1. BCR-ABL1 γH2AX regions summary, related to Figure 1 and 2. This table contains the lists of annotated γH2AX regions identified by SICER for BCR-ABL1-transformed B-cell precursors. Shown are the normalized reads per million (RPM; isotype background subtracted), fold change, reads per kilo base per million (RPKM) and gene annotations of regions enriched for the γH2AX ChIP-seq signal versus the isotype control library.

Table S2. C-MYC γH2AX regions summary, related to Figure 1 and 2. This table contains the lists of annotated γH2AX regions identified by SICER for C-MYC-transformed B-cell precursors. Shown are the normalized reads per million (RPM; isotype background subtracted), fold change, reads per kilo base per million (RPKM) and gene annotations of regions enriched for the γH2AX ChIP-seq signal versus the isotype control library.

Table S3. Merged γH2AX regions summary, related to Figure 1, 4 and 5. This table contains the merged list of annotated γH2AX regions. Shown are the annotations and chromosomal locations of γH2AX enriched regions present in BCR-ABL1-transformed B-cell precursors, C-MYC-transformed B-cell precursors or in both as indicated.

Table S4. Merged γH2AX regions GO results, related to Figure S2D. This table contains the output of the Gene Ontology (GO) analysis of γH2AX regions. Shown are the enriched biological process (BP) gene ontology (GO) categories for genes associated with the merged γH2AX enriched regions indicated by Table S3.

Table S5. Gene defects in human B-ALL vs. γH2AX regions, related to Figure 3. The table shows the comparisons of genes associated with γH2AX-enriched regions in transformed B-cell precursors from this study to genes associated with genomic deletions or translocations in human B-ALL identified by Mullighan et al., 2007, Mullighan et al., 2009 or Roberts et al., 2012 as indicated. Data used for comparison relates to the information provided by the supplementary information of these studies, as indicated in this table and the Supplemental Experimental Procedures section.

Table S6. CEBPA+DOX specific γH2AX regions summary, related to Figure 6. This table contains the lists of annotated γH2AX regions identified by SICER as specific for myeloid-reprogrammed BCR-ABL1-transformed B-cell precursors. Shown are fold change, reads per kilo base per million (RPKM) and gene annotations of regions enriched for the γH2AX ChIP-seq signal in DOX-treated vs. untreated *CEBPA*-transgenic BCR-ABL1-transformed B-cell precursors.

Table S7. CEBPA+DOX specific γH2AX regions GO results, related to Figure 6. This table contains the output of the Gene Ontology (GO) analysis of γH2AX regions identified by SICER as specific for myeloid-reprogrammed BCR-ABL1-transformed B-cell precursors. Shown are the enriched biological process (BP) gene ontology (GO) categories for genes associated with the γH2AX enriched regions in DOX-treated vs. untreated *CEBPA*-transgenic BCR-ABL1 transformed B-cell precursors.

Supplemental Experimental Procedures

Chromatin immunoprecipitation (ChIP): Ex-vivo cultured B-cell precursor cells were prepared for ChIP-seq analysis according to (Yamane et al., 2013) with minor modifications: All cells used for ChIP-seq were depleted of dead/apoptotic cells using the dead cell removal kit (Miltenyi). Formaldehyde used for crosslinking was from Alfa Aesar and cells were snap-frozen as cell pellets before start of the ChIP procedure. Before sonication, cell pellets were re-suspended in 130μL of sonication buffer (0.5% SDS, 10mM Tris-HCl pH7.6) supplemented with protease and phosphatase inhibitor (Roche) and incubated for 20 minutes on ice. Sonication was performed using microTUBE AFA 6x16mm on the Covaris S220 sonicator and the following settings to yield an average DNA fragment size of 300bp: peak power 105, duty factor 5 and cycles/burst 200 for 5 minutes. Sonicated cells were transferred to concentrated RIPA buffer (10mM Tris-HCl pH7.6, 1.15mM EDTA, 0.04% SDS, 1.15% TritonX-100, 0.115% sodium deoxycholate) to yield 1ml of 1X RIPA buffer described in (Yamane et al., 2013), and subjected to ChIP using 5μg ab/12-15x10^6 cells of the following antibodies: Anti-gamma H2A.X (phospho S139) antibody - ChIP Grade (ab2893) (Abcam), Anti-Histone H3 (acetyl K27) antibody – ChIP Grade (ab4729) (Abcam), polyclonal rabbit IgG (isotype control) (sc2027) (Santa Cruz Biotechnologies). All sonications were verified by agarose gel electrophoresis, and respective immunoprecipitations controlled by Western blot. ChIP pull-down DNA was quantified using the Quant-iT™ PicoGreen® dsDNA Assay Kit (Thermo Fisher).

ChIP-Seq library preparation: ChIP-seq libraries were generated from ChIP pull-down DNA or from input DNA of the same ChIP procedure in parallel as control using the NEBNext® ChIP-seq Library Prep Master Mix Set and NEBNext Multiplex Oligos for Illumina (NEB). Pre-amplified ChIP-seq libraries were then sequenced by the genomics facilities of the Medical Research Council (MRC) and Imperial College London (ICL).

ChIP-Seq libraries: In total, 15 ChIP libraries were generated: Two repeats of γH2AX-ChIP-seq from two individual and time-separated experiments for BCR-ABL1 and C-MYC transduced and IL7 cultured B-cell precursors harvested on day 7 post oncogene induction. Respective control libraries using isotype control antibodies for ChIP or input DNA were generated in parallel. In addition, one γH2AX-ChIP-seq library from long-term cultured (>D50) BCR-ABL1-transformed B-cell precursors was generated in parallel. These >D50 cells had been additionally treated for 2 hours with 10mM Hydroxyurea before harvest. For H3K27ac analysis four ChIP-seq libraries have been generated: One H3K27ac-ChIP-seq library each for BCR-ABL1 and C-MYC transduced IL7 cultured B-cell precursors harvested on day 7 post oncogene induction, and two respective input DNA control libraries. Five additional ChIP-seq libraries were generated on BCR-ABL1-transformed B-cell precursors (CEBPA experiment, Figure 6) cultured in the absence of IL7 and originally obtained from Markus Müschen laboratory (Chen et al., 2015). These include one library each for cells carrying an inducible CEBPA transgene treated either with Doxycycline or not, and one library for control cells carrying the inducible vector without CEBPA (empty vector, EV) and treated with Doxycycline. For CEBPA +DOX and EV +DOX conditions input DNA control libraries were generated.

ChIP-Seq read processing and generation of custom tracks: Sequence reads of 50 bps were obtained through standard Illumina image-analysis and default quality filters. Reads were aligned to the Build 38 mouse genome assembly (GRCm38/mm10) using BWA-MEM with default settings. Non-uniquely aligned reads were removed using SAMtools (Li and Durbin, 2009) and PCR duplicates were removed using Picard tools (http://broadinstitute.github.io/picard). To generate custom tracks for visualization, coverage within 200 bp windows was calculated and normalized to library and window size using deepTools (Ramirez et al., 2014) presented as reads per kilobase per million mapped reads (RPKM).

ChIP-Seq read enriched region calling: Region calling was done as described in (Barlow et al., 2013) with minor modifications to further optimize calling of γH2AX-enriched regions. In brief, sequence reads were pre-processed as described above and statistical enrichment of uniquely aligned reads mapping within non-overlapping 100bp windows was analyzed relative to a Poissonian random background using the SICER program (Zang et al., 2009) with parameters: e-value 10,000, gap size 300. Statistical enrichment of bins passing the first criterion was examined relative to negative control DNA from either input or isotype control ChIP reactions based on tag density fold increase determined from False Discovery Rate of 1 x 10^{-5}. Subsequently, identified enriched windows that were less than 10 kb apart were merged to delineate enriched genomic regions. Finally, an empirical filtering step was applied to eliminate any regions with low coverage (<1.5 RPKM), width (<5 kb) or

fold change (< 1.1), resulting in the final lists of highly enriched regions. While these settings were optimized for calling of γH2AX-enriched regions, the same settings were used for H3K27ac enriched region calling.

Generation of a merged γH2AX-enriched region list: Stringent lists of γH2AX-enriched regions from BCR-ABL1 and C-MYC were merged using the GRanges package (Lawrence et al., 2013) in R (R Core Team, 2015; https://www.R-project.org), taking the union of any overlapping regions.

Chromosomal distribution of enriched regions: RPM values for γH2AX-enriched regions from the merged stringent list were calculated using BEDTools (Quinlan and Hall, 2010). Ideograms in Figure 1F were plotted using the IdeoViz package in R and RPM values from BCR-ABL1 (run1). The percentage of total chromosome coverage for the merged region list shown in Figure 1G was calculated using Granges (Lawrence et al., 2013) and plotted against exon coverage estimates obtained from (Sakharkar et al., 2005).

Correlation of γH2AX signals from different ChIP-Seq libraries: RPM values for γH2AX-enriched regions from the merged stringent list were calculated using BEDTools (Quinlan and Hall, 2010) for BCR-ABL1 and C-MYC samples, with subtraction of the isotype signal. Scatter plots of log2 signal intensity were generated and overlapping/unique regions highlighted. Linear regression fit (red line) and Pearson's rho values (r) were calculated for each comparison.

Analysis of enriched regions for gene ontology (GO) pathway enrichment: Genes overlapping γH2AX-enriched regions from BCR-ABL1 and C-MYC samples were identified using the R package ChIPpeakAnno (Zhu et al., 2010) and enriched biological process GO categories identified (P < 0.01). Enriched GO terms were associated with four empirically chosen parent categories: chromosome organization (GO:0051276), gene expression (GO:0010467), nucleic acid metabolic process (GO:0090304) and immune system process (GO:0002376). Pie charts were plotted for each enriched set before identifying common categories between BCR-ABL1 and C-MYC samples, removal of redundant GO terms and plotting of -log10 p-values.

Genic distribution of enriched regions: Meta-gene profiles of γH2AX and H3K27ac signal distribution in Inset of Figure 4A and in Figure S4B were plotted for all Ensembl protein-coding genes (n=22,533) plus 10kb up- and down-stream using the ngs.plot package (Shen et al., 2014). Signal profiles for all meta-gene profiles are normalized to gene length by spline fitting, to library size by RPM, and plotted as log2 fold change over isotype control ChIP-seq library. The intersection of enriched regions overlapping genic features shown in Figure 4A was determined using the ChIPSeeker R package (Yu et al., 2015) with TSS defined as +/- 1kb and default settings.

Correlation of γH2AX and H3K27ac signals: Stringent region lists for both H3K27ac and γH2AX were merged for BCR-ABL1 and C-MYC, and RPM values within these regions were calculated from BCR-ABL1, C-MYC and isotype control samples using BEDTools before performing background subtraction. Negative signal values were arbitrarily set to 0.01 RPM for visualization purposes and excluded during calculation of Pearson's correlation coefficients. Scatter plots of log10 signal intensity were generated and overlapping/unique regions highlighted. Red lines indicate the linear regression fit.

Comparison of γH2AX regions to location of ERFS: Identified γH2AX regions were compared to a previously published ChIP-seq dataset that characterized early replicating fragile sites (ERFS) in Hydroxyurea-treated mature B-cells (Barlow et al., 2013) (region list: Table S1, accession number: SRR648771). Published ERFS regions were converted from NCBI37/mm9 to GRCm38/mm10 (UCSC mm9ToMm10 chain file) for comparison to mm10 referenced γH2AX regions. FASTQ reads were extracted from SRA files using the SRA-toolkit and aligned against the mm10 genome as detailed for our own data. The observed overlap between regions was compared against the average number of permuted overlaps. Permutation models were performed with sex chromosomes and unmapable regions masked for 100,000 enumerations.

Comparison of γH2AX regions to location of RLFS: Locations of potential R-loop forming sequences (RLFS) were identified throughout the mm10 genome using QmRLFS-finder (Jenjaroenpun et al., 2015). Permutation testing was performed with the R package regioneR (Gel et al., 2016) with 10,000 enumerations per test. Analysis of RLFS enrichment within γH2AX regions was determined by re-sampling of genomic regions within masked mm10 genome, while enrichment within genes was determined through re-sampling of UCSC known-

genes. Enrichment of RLFS within highly expressed genes was performed in a similar manner but first restricting the total gene population to the top 1000 most high-expressed genes as ranked by mean expression across all conditions analyzed by RNA-seq.

Comparison of γH2AX regions to genomic defects in leukemia: For comparison of gene sets as shown in Figure 3B, datasets published in Mullighan et al 2007 (Mullighan et al., 2007) (Table S10 in respective article), Mullighan et al 2009 (Mullighan et al., 2009) (Table S4 in respective article) and Roberts et al 2012 (Roberts et al., 2012) (Table S3 and S7 in respective article) were used. Only defects that were reported for B-ALL and that relate to genomic deletion or translocation were used for comparison. Further, only those defects could be compared that were indicated to relate to annotated genes, and for which a respective counterpart could be found in the mouse genome. For example, iAmp21, Znf528, Ccdc26, Fam22f, C20orf94 and FLJ11273 from the Mullighan datasets were excluded from analysis because we could not relate them to a unique mouse gene. Also, lesions of extreme large sizes were not included into the analysis (e.g. 'all 10p' or '383 genes involved'). Reported defects affecting multiple genes (e.g. Atm/Rab39/Npat) were counted as one defect, and only one overlap of γH2AX regions was counted even if multiple genes within the defect exhibited overlap with respective γH2AX regions. This lead to a list of 39 alterations for comparison for the Mullighan et al 2007 dataset, 42 alterations for comparison for the Mullighan et al 2009 dataset, and 42 alterations for comparison for the Roberts et al 2012 dataset as indicated in detail in Supplemental Table S5.

Analysis of genic vs. intergenic distribution of structural variant (SV) breakpoints in leukemia genomes: To analyze the genic vs. intergenic distribution of SV breakpoints identified in leukemia genomes as shown in Figure S4A, published breakpoint coordinates from Mullighan et al 2007 (Mullighan et al., 2007) (Table S10 in respective article), Papaemmanuil et al 2014 (Papaemmanuil et al., 2014) (Table S4 in respective article), Andersson et al 2015 (Andersson et al., 2015) (Table S7 in respective article), Holmfeldt et al 2013 (Holmfeldt et al., 2013) (Table S8 in respective article), and Paulsson et al 2015 (Paulsson et al., 2015) (Table S11 in respective article) were used. SV breakpoint coordinates were mapped to gene regions in R (exon, intron, promoter = 3kb upstream, downstream = 3kb downstream, distal = >3kb from gene boundaries). Significance of enrichment in genic regions was tested by hypergeomtric distribution test.

Comparison of γH2AX regions to replication timing: γH2AX regions were categorized as early or late replicating by comparison with ENCODE Repli-chip data (Mouse et al., 2012). A zero-crossing algorithm was applied to wavelet-smoothed Repli-chip data from the CH12 cell line to identify early-replicating regions, and only regions called as early replicating in both biological replicates used to annotate γH2AX regions. Permutation models were performed as described previously.

RNA-Seq analysis: Reads were aligned to the mouse genome (GRCm38/mm10) using STAR v2.5.0 (Dobin et al., 2013), indexes were supplemented with UCSC known gene reference transcript assemblies. Alignment was performed with the following settings: --outFilterType BySJout, --outFilterMultimanNmax 20, --alignSJoverhangMin 8, --alignSJDBoverhangMin 1, --outFilterMismatchNmax 999, --outFilterMismatchNoverLmax 0.04, --alignIntronMin 20, --alignIntronMax 1000000. FPKM values were calculated with StringTie v1.3.0 (Pertea et al., 2015). Differential expression analysis was performed in R (R Core Team, 2016; https://www.R-project.org) with DESeq2 v1.12.4 (Love et al., 2014).

Comparison of RNA-Seq and γH2AX-ChIP-seq data: All UCSC known genes were categorized into low or high expression groups relative to the median FPKM value of expressed genes for each condition. The proportion of genes overlapping γH2AX regions falling into high or low expression categories was then determined. To compare γH2AX signals in high vs. low expression categories and in BCR-ABL1 vs. C-MYC differentially expressed comparison, all UCSC-known gene regions were extended by 10 kb and the γH2AX signal within these regions determined using the R package bamsignals v1.4.3, normalized to library size, and background signal from isotype control libraries subtracted.

DS-RED ChIP-qPCR: For analysis of DNA damage upon inducible expression of the fluorochrome DS-RED, the expression vector TRMPVIR (Addgene) was modified by digestion with EcoRV and XhoI to remove the mVenus and rtTA cassettes. MYC induced B-cell precursor cells used for the experiments were generated from *53BP1*-/- mice and first transduced with the TET3G expression plasmid (Addgene) followed by selection using 500µg/mL Geneticin/G418 (Sigma). Pre-selected cells were then transduced in triplicate with 24h time between

each round of transduction using retrovirus encoding the modified TRMPVIR vector. Three days after the last transduction, 1μg/mL DOX was added to the media. After three days of DOX treatment cells were harvested for analysis by ChIP-qPCR. Cells were processed similar to cells designated for ChIP-seq including analysis by flow cytometry and dead cell removal by MACS. ChIP DNA was analysed in triplicate using SYBR Green Jumpstart Taq ReadyMix (Sigma) on the StepOnePlus (Applied Biosciences). For quantification of DS-RED the following primers were used: CGAGTTCATGCGCTTCAAGG as forward primer and GTCACCTTCAGCTTCACGGT as reverse primer.

qRT-PCR. For IL-7 cultured control and transformed cells, cells were harvested at day 7 p.i. as described, dead cells were removed by dead cell removal kit (MACS, Miltenyi), and then snap frozen on dry ice. For thymus and BM, cells were isolated freshly from *53BP1*$^{-/-}$ mice and snap frozen on dry ice. Stimulated B-cells were obtained freshly from *53BP1*$^{-/-}$ mice and cultured as previously described (Feldhahn et al., 2012). RNA was isolated from cells using a column-based kit (Qiagen RNeasy) and cDNA was generated using RevertAid cDNA Synthesis Kit (Thermo Fisher). qPCR was performed using a SYBR green master mix (Sigma JumpStart) on a StepOnePlus platform (Thermo Fisher) and the following primer pairs: RAG1 forward AGGCCTGTGGAGCAAGGTAG and reverse TTTCATCGGGTGCAGAACTGA at 63ºC annealing; AID forward CCCTTGTACGAAGTCGATGAC and reverse ATCACGTGTGACATTCCAGGAG at 60ºC annealing (Crouch et al., 2007) HPRT forward CCCAGCGTCGTGATTAGC and reverse GGAATAAACACTTTTTCCAAAT at 60ºC annealing. Reactions were performed in triplicate and the average Ct and reaction efficiency were analyzed using the Pfaffl method (Pfaffl, 2001) to determine relative expression of AID and RAG1 using HPRT as the endogenous control. Expression levels of genes in experimental cells were plotted relative to expression in resting, naïve splenic B-cells.

Supplemental References

Andersson, A.K., Ma, J., Wang, J., Chen, X., Gedman, A.L., Dang, J., Nakitandwe, J., Holmfeldt, L., Parker, M., Easton, J.*, et al.* (2015). The landscape of somatic mutations in infant MLL-rearranged acute lymphoblastic leukemias. Nat Genet *47*, 330-337.

Barlow, J.H., Faryabi, R.B., Callen, E., Wong, N., Malhowski, A., Chen, H.T., Gutierrez-Cruz, G., Sun, H.W., McKinnon, P., Wright, G.*, et al.* (2013). Identification of early replicating fragile sites that contribute to genome instability. Cell *152*, 620-632.

Berger, M., Krebs, P., Crozat, K., Li, X., Croker, B.A., Siggs, O.M., Popkin, D., Du, X., Lawson, B.R., Theofilopoulos, A.N.*, et al.* (2010). An Slfn2 mutation causes lymphoid and myeloid immunodeficiency due to loss of immune cell quiescence. Nat Immunol *11*, 335-343.

Chen, Z., Shojaee, S., Buchner, M., Geng, H., Lee, J.W., Klemm, L., Titz, B., Graeber, T.G., Park, E., Tan, Y.X.*, et al.* (2015). Signalling thresholds and negative B-cell selection in acute lymphoblastic leukaemia. Nature *521*, 357-361.

Clausen, B.E., Burkhardt, C., Reith, W., Renkawitz, R., and Forster, I. (1999). Conditional gene targeting in macrophages and granulocytes using LysMcre mice. Transgenic Res *8*, 265-277.

Crouch, E.E., Li, Z., Takizawa, M., Fichtner-Feigl, S., Gourzi, P., Montano, C., Feigenbaum, L., Wilson, P., Janz, S., Papavasiliou, F.N.*, et al.* (2007). Regulation of AID expression in the immune response. J Exp Med *204*, 1145-1156.

Dobin, A., Davis, C.A., Schlesinger, F., Drenkow, J., Zaleski, C., Jha, S., Batut, P., Chaisson, M., and Gingeras, T.R. (2013). STAR: ultrafast universal RNA-seq aligner. Bioinformatics *29*, 15-21.

Feingold, K.R., Kazemi, M.R., Magra, A.L., McDonald, C.M., Chui, L.G., Shigenaga, J.K., Patzek, S.M., Chan, Z.W., Londos, C., and Grunfeld, C. (2010). ADRP/ADFP and Mal1 expression are increased in macrophages treated with TLR agonists. Atherosclerosis *209*, 81-88.

Feldhahn, N., Ferretti, E., Robbiani, D.F., Callen, E., Deroubaix, S., Selleri, L., Nussenzweig, A., and Nussenzweig, M.C. (2012). The hSSB1 orthologue Obfc2b is essential for skeletogenesis but dispensable for the DNA damage response in vivo. EMBO J *31*, 4045-4056.

Gel, B., Diez-Villanueva, A., Serra, E., Buschbeck, M., Peinado, M.A., and Malinverni, R. (2016). regioneR: an R/Bioconductor package for the association analysis of genomic regions based on permutation tests. Bioinformatics *32*, 289-291.

Holmfeldt, L., Wei, L., Diaz-Flores, E., Walsh, M., Zhang, J., Ding, L., Payne-Turner, D., Churchman, M., Andersson, A., Chen, S.C.*, et al.* (2013). The genomic landscape of hypodiploid acute lymphoblastic leukemia. Nat Genet *45*, 242-252.

Jenjaroenpun, P., Wongsurawat, T., Yenamandra, S.P., and Kuznetsov, V.A. (2015). QmRLFS-finder: a model, web server and stand-alone tool for prediction and analysis of R-loop forming sequences. Nucleic Acids Res *43*, W527-534.

Kim, Y., Schulz, V.P., Satake, N., Gruber, T.A., Teixeira, A.M., Halene, S., Gallagher, P.G., and Krause, D.S. (2014). Whole-exome sequencing identifies a novel somatic mutation in MMP8 associated with a t(1;22)-acute megakaryoblastic leukemia. Leukemia *28*, 945-948.

Lawrence, M., Huber, W., Pages, H., Aboyoun, P., Carlson, M., Gentleman, R., Morgan, M.T., and Carey, V.J. (2013). Software for computing and annotating genomic ranges. PLoS computational biology *9*, e1003118.

Li, H., and Durbin, R. (2009). Fast and accurate short read alignment with Burrows-Wheeler transform. Bioinformatics (Oxford, England) *25*, 1754-1760.

Love, M.I., Huber, W., and Anders, S. (2014). Moderated estimation of fold change and dispersion for RNA-seq data with DESeq2. Genome biology *15*, 550.

Mouse, E.C., Stamatoyannopoulos, J.A., Snyder, M., Hardison, R., Ren, B., Gingeras, T., Gilbert, D.M., Groudine, M., Bender, M., Kaul, R*., et al.* (2012). An encyclopedia of mouse DNA elements (Mouse ENCODE). Genome biology *13*, 418.

Mullighan, C.G., Goorha, S., Radtke, I., Miller, C.B., Coustan-Smith, E., Dalton, J.D., Girtman, K., Mathew, S., Ma, J., Pounds, S.B*., et al.* (2007). Genome-wide analysis of genetic alterations in acute lymphoblastic leukaemia. Nature *446*, 758-764.

Mullighan, C.G., Su, X., Zhang, J., Radtke, I., Phillips, L.A., Miller, C.B., Ma, J., Liu, W., Cheng, C., Schulman, B.A*., et al.* (2009). Deletion of IKZF1 and prognosis in acute lymphoblastic leukemia. N Engl J Med *360*, 470-480.

Papaemmanuil, E., Rapado, I., Li, Y., Potter, N.E., Wedge, D.C., Tubio, J., Alexandrov, L.B., Van Loo, P., Cooke, S.L., Marshall, J*., et al.* (2014). RAG-mediated recombination is the predominant driver of oncogenic rearrangement in ETV6-RUNX1 acute lymphoblastic leukemia. Nat Genet *46*, 116-125.

Park, Y.J., Yoon, S.J., Suh, H.W., Kim, D.O., Park, J.R., Jung, H., Kim, T.D., Yoon, S.R., Min, J.K., Na, H.J*., et al.* (2013). TXNIP deficiency exacerbates endotoxic shock via the induction of excessive nitric oxide synthesis. PLoS Pathog *9*, e1003646.

Paul, S.P., Taylor, L.S., Stansbury, E.K., and McVicar, D.W. (2000). Myeloid specific human CD33 is an inhibitory receptor with differential ITIM function in recruiting the phosphatases SHP-1 and SHP-2. Blood *96*, 483-490.

Paulsson, K., Lilljebjorn, H., Biloglav, A., Olsson, L., Rissler, M., Castor, A., Barbany, G., Fogelstrand, L., Nordgren, A., Sjogren, H*., et al.* (2015). The genomic landscape of high hyperdiploid childhood acute lymphoblastic leukemia. Nat Genet *47*, 672-676.

Pertea, M., Pertea, G.M., Antonescu, C.M., Chang, T.C., Mendell, J.T., and Salzberg, S.L. (2015). StringTie enables improved reconstruction of a transcriptome from RNA-seq reads. Nat Biotechnol *33*, 290-295.

Pfaffl, M.W. (2001). A new mathematical model for relative quantification in real-time RT-PCR. Nucleic acids research *29*, e45.

Quinlan, A.R., and Hall, I.M. (2010). BEDTools: a flexible suite of utilities for comparing genomic features. Bioinformatics *26*, 841-842.

Ramirez, F., Dundar, F., Diehl, S., Gruning, B.A., and Manke, T. (2014). deepTools: a flexible platform for exploring deep-sequencing data. Nucleic acids research *42*, W187-191.

Roberts, K.G., Morin, R.D., Zhang, J., Hirst, M., Zhao, Y., Su, X., Chen, S.C., Payne-Turner, D., Churchman, M.L., Harvey, R.C*., et al.* (2012). Genetic alterations activating kinase and cytokine receptor signaling in high-risk acute lymphoblastic leukemia. Cancer Cell *22*, 153-166.

Sakharkar, M.K., Perumal, B.S., Sakharkar, K.R., and Kangueane, P. (2005). An analysis on gene architecture in human and mouse genomes. In silico biology *5*, 347-365.

Shen, L., Shao, N., Liu, X., and Nestler, E. (2014). ngs.plot: Quick mining and visualization of next-generation sequencing data by integrating genomic databases. BMC genomics *15*, 284.

Veillette, A., Thibaudeau, E., and Latour, S. (1998). High expression of inhibitory receptor SHPS-1 and its association with protein-tyrosine phosphatase SHP-1 in macrophages. J Biol Chem *273*, 22719-22728.

Yamane, A., Robbiani, D.F., Resch, W., Bothmer, A., Nakahashi, H., Oliveira, T., Rommel, P.C., Brown, E.J., Nussenzweig, A., Nussenzweig, M.C.*, et al.* (2013). RPA accumulation during class switch recombination represents 5'-3' DNA-end resection during the S-G2/M phase of the cell cycle. Cell reports *3*, 138-147.

Yokoyama, T., Kanno, Y., Yamazaki, Y., Takahara, T., Miyata, S., and Nakamura, T. (2010). Trib1 links the MEK1/ERK pathway in myeloid leukemogenesis. Blood *116*, 2768-2775.

Yu, G., Wang, L.G., and He, Q.Y. (2015). ChIPseeker: an R/Bioconductor package for ChIP peak annotation, comparison and visualization. Bioinformatics *31*, 2382-2383.

Zang, C., Schones, D.E., Zeng, C., Cui, K., Zhao, K., and Peng, W. (2009). A clustering approach for identification of enriched domains from histone modification ChIP-Seq data. Bioinformatics *25*, 1952-1958.

Zhao, Y., Xiong, Z., Lechner, E.J., Klenotic, P.A., Hamburg, B.J., Hulver, M., Khare, A., Oriss, T., Mangalmurti, N., Chan, Y.*, et al.* (2014). Thrombospondin-1 triggers macrophage IL-10 production and promotes resolution of experimental lung injury. Mucosal Immunol *7*, 440-448.

Zhou, X., Sun, L., Bastos de Oliveira, F., Qi, X., Brown, W.J., Smolka, M.B., Sun, Y., and Hu, F. (2015). Prosaposin facilitates sortilin-independent lysosomal trafficking of progranulin. J Cell Biol *210*, 991-1002.

Zhu, L.J., Gazin, C., Lawson, N.D., Pages, H., Lin, S.M., Lapointe, D.S., and Green, M.R. (2010). ChIPpeakAnno: a Bioconductor package to annotate ChIP-seq and ChIP-chip data. BMC bioinformatics *11*, 237.
